# Genome-Wide Negative Feedback Drives Transgenerational DNA Methylation Dynamics in Arabidopsis

**DOI:** 10.1371/journal.pgen.1005154

**Published:** 2015-04-22

**Authors:** Tasuku Ito, Yoshiaki Tarutani, Taiko Kim To, Mohamed Kassam, Evelyne Duvernois-Berthet, Sandra Cortijo, Kazuya Takashima, Hidetoshi Saze, Atsushi Toyoda, Asao Fujiyama, Vincent Colot, Tetsuji Kakutani

**Affiliations:** 1 Department of Integrated Genetics, National Institute of Genetics, Mishima, Shizuoka, Japan; 2 Department of Biological Sciences, Graduate School of Science, The University of Tokyo, Hongo, Bunkyo-ku, Tokyo, Japan; 3 Department of Genetics, School of Life Science, The Graduate University for Advanced Studies (SOKENDAI), Yata, Shizuoka, Japan; 4 Ecole Normale Supérieure, Institut de Biologie (IBENS), Centre National de la Recherche Scientifique (CNRS) UMR8197, Institut National de la Santé et de la Recherche Médicale (INSERM) U1024, Paris, France; 5 Okinawa Institute of Science and Technology, Onna-son, Okinawa, Japan; 6 Center for Information Biology, National Institute of Genetics, Yata, Shizuoka, Japan; Gregor Mendel Institute of Molecular Plant Biology, AUSTRIA

## Abstract

Epigenetic variations of phenotypes, especially those associated with DNA methylation, are often inherited over multiple generations in plants. The active and inactive chromatin states are heritable and can be maintained or even be amplified by positive feedback in a transgenerational manner. However, mechanisms controlling the transgenerational DNA methylation dynamics are largely unknown. As an approach to understand the transgenerational dynamics, we examined long-term effect of impaired DNA methylation in Arabidopsis mutants of the chromatin remodeler gene *DDM1* (*Decrease in DNA Methylation 1*) through whole genome DNA methylation sequencing. The *ddm1* mutation induces a drastic decrease in DNA methylation of transposable elements (TEs) and repeats in the initial generation, while also inducing ectopic DNA methylation at hundreds of loci. Unexpectedly, this ectopic methylation can only be seen after repeated self-pollination. The ectopic cytosine methylation is found primarily in the non-CG context and starts from 3’ regions within transcription units and spreads upstream. Remarkably, when chromosomes with reduced DNA methylation were introduced from a *ddm1* mutant into a *DDM1* wild-type background, the *ddm1*-derived chromosomes also induced analogous de novo accumulation of DNA methylation in trans. These results lead us to propose a model to explain the transgenerational DNA methylation redistribution by genome-wide negative feedback. The global negative feedback, together with local positive feedback, would ensure robust and balanced differentiation of chromatin states within the genome.

## Author Summary

DNA methylation is important for controlling activity of transposable elements and genes. An intriguing feature of DNA methylation in plants is that its pattern can be inherited over multiple generations at high fidelity in a Mendelian manner. However, mechanisms controlling the trans-generational DNA methylation dynamics are largely unknown. Arabidopsis mutants of a chromatin remodeler gene *DDM1* (*Decrease in DNA Methylation 1*) show drastic reduction of DNA methylation in transposons and repeats, and also show progressive changes in developmental phenotypes during propagation through self-pollination. We now show using whole genome DNA methylation sequencing that upon repeated selfing, the *ddm1* mutation induces an ectopic accumulation of DNA methylation at hundreds of loci. Remarkably, even in the wild type background, the analogous de novo increase of DNA methylation can be induced in trans by chromosomes with reduced DNA methylation. Collectively, our findings support a model to explain the transgenerational DNA methylation redistribution by genome-wide negative feedback, which should be important for balanced differentiation of DNA methylation states within the genome.

## Introduction

Epigenetic variation of gene expression is mediated by chromatin marks, such as modifications of histones and DNA. Importantly, these marks and associated gene expression patterns can be inherited over multiple generations in both animals and plants [1,2]. Transgenerational epigenetic inheritance, especially the one associated with DNA methylation, is widespread in plants, and that could have a significant impact on evolution [3–5]. The long-term dynamics of DNA methylation has recently been examined genome-wide at single base resolution in the flowering plant Arabidopsis [6,7]; by analysing repeatedly self-pollinated wild type Arabidopsis plants, heritable gain and loss of DNA methylation have been detected, although their frequencies are generally low. A complementary approach to uncover the background mechanisms controlling long-term DNA methylation dynamics is to examine the effects of impaired DNA methylation pattern over multiple generations.

Factors controlling genomic DNA methylation have been studied extensively in Arabidopsis; and many of these factors constitute positive feedback loops to stabilize epigenetic states. Cytosine methylation in the context of dinucleotide CG is maintained by maintenance methyltransferase MET1 [8,9], while cytosine methylation at non-CG site is mediated by chromomethylases (CMTs) [10,11]. The CMTs are recruited to chromatin by methylation of histone H3 lysine 9 (H3K9me), and the H3K9 methylase KYP/SUH4 is also recruited to chromatin with non-CG methylation, generating a self-reinforcing positive feedback loop [11–14]. Both H3K9me and non-CG methylation are silent heterochromatin marks normally found in repeats and transposable elements (TEs); and these marks are rarely detectable in transcribed genes. Exclusion of these marks from transcribed genes depends on the H3K9 demethylase IBM1 (Increase in BONSAI Methylation 1) [13,15]. IBM1 removes H3K9me from transcribed genes, generating another positive feedback loop to stabilize active states [13]. In addition, a positive feedback loop is also found in a process called RNA-directed DNA methylation (RdDM). RdDM is a de novo DNA methylation process triggered by double-strand RNA; and factors involved in this process have been extensively studied [16–20]. The final step of RdDM is DNA methylation of both CG and non-CG sites by the de novo DNA methyltransferase DRM2 (Domains Rearranged Methylase 2), with the RNAi machinery and small interfering RNA (siRNA) functioning as upstream factors. Interestingly, production of siRNA also depends on *DRM2* [21,22], suggesting another positive feedback that stabilizes the silent state. Genome-wide DNA methylation profiles have been determined in mutants of these and other factors controlling DNA methylation [11,23,24], although information for the transgenerational effects of these mutations is limited.

Among the Arabidopsis mutants affecting genomic DNA methylation, *ddm1* (decrease in DNA methylation 1) is one of the mutations with the strongest effects. Mutant plants show drastic reduction of DNA methylation at both CG and non-CG sites in heterochromatic repeats and TEs [25,26]. The *DDM1* gene encodes a chromatin remodeling factor, which is necessary for DNA methylation in heterochromatic sequences [10,27]. Mutation in its mammalian ortholog *Lsh* induces loss of DNA methylation, suggesting conserved functions across the animal and plant kingdoms [28,29].

A striking feature of the Arabidopsis *ddm1* mutant is the progressive accumulation of the developmental defects; initial generations of the *ddm1* mutant grow relatively normally, but many types of developmental abnormalities arise after multiple rounds of self-pollinations [30,31]. Some of the abnormalities are due to DNA sequence changes, such as insertion mutations of de-repressed endogenous TEs [32–34] or a rearrangement of repeats [35], but others are due to epigenetic changes in gene expression, which correlate with changes in DNA methylation pattern at the affected loci [36,37].

Here we analyze the transgenerational effects of the *ddm1* mutation genome-wide, by comparing DNA methylation of the *ddm1* mutants before and after the repeated self-pollinations. This analysis revealed ectopic accumulation of non-CG methylation at hundreds of loci; and unexpectedly, this hypermethylation could only be seen after repeated self-pollinations. Furthermore, when *ddm1*-derived chromosomes with disrupted heterochromatin were introduced into a *DDM1* wild type background, de novo accumulation of non-CG methylation was induced in trans. These results lead us to propose a model in which loss of heterochromatin is progressively compensated for through a negative feedback mechanism that leads to heterochromatin redistribution across the genome.

## Results

### Early and late generations of *ddm1* mutants show distinct genomic DNA methylation patterns

**Fig 1:**
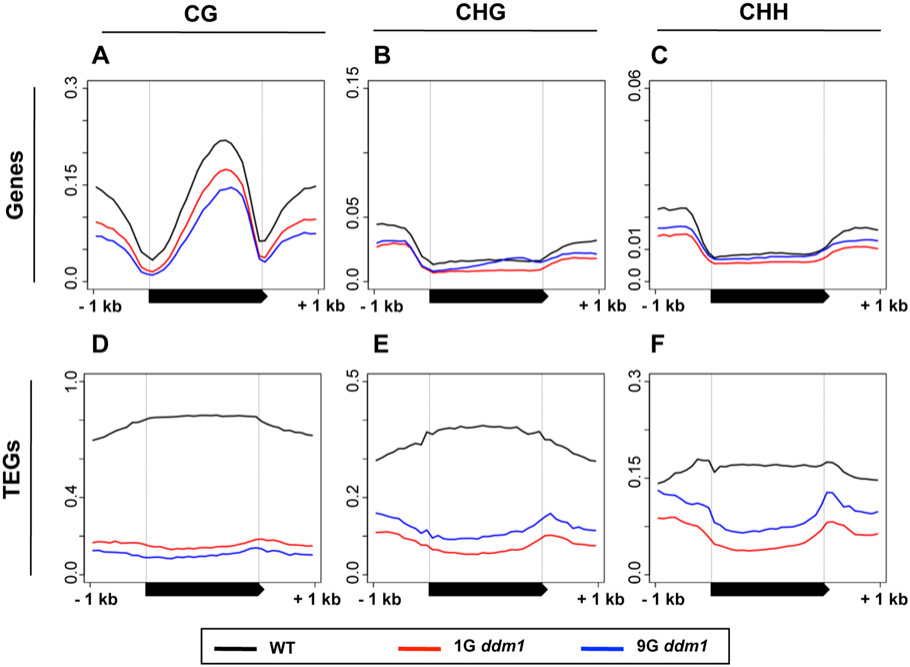
DNA methylation in *ddm1* mutants before and after repeated self-pollination. DNA methylation patterns of WT, 1G *ddm1*, and 9G *ddm1* mutants for cellular genes (A-C) or genes within transposable elements (transposable element genes, or TEGs, D-F). “WT” is a *DDM1/DDM1* plant segregating as a sibling of the 1G *ddm1/ddm1* plants. The black bars in the bottom represent transcribed regions. A chromosome-wide view of DNA methylation is also shown in S2 Fig.

To understand the changes in DNA methylation patterns during self-pollinations of *ddm1* mutant genome-wide, we compared DNA methylation before and after the self-pollination of the mutant. We examined DNA methylation in four individuals of *ddm1* homozygous mutants segregated in progeny of a heterozygote (hereafter called 1G for the 1st Generation) and also four lines of *ddm1* plants independently self-pollinated eight times (hereafter called 9G) (S1 Fig). In 1G, the *ddm1* mutation already induced reduction of DNA methylation in heterochromatic regions [10,25,26]. Methylation in repetitive sequences, such as transposable elements (TEs) (Fig 1D–1F), was much more severely affected than that in low copy sequences, such as genes (Fig 1A–1C). The reduction was found for both CG sites (Fig 1A and 1D) and non-CG sites. In non-CG sites, both CHG sites (Fig 1B and 1E) and CHH sites (Fig 1C and 1F) were affected (H can be A, T, or C). When we compared average DNA methylation of 9G to 1G, two features were noted for both genes and TEs: further decrease of CG methylation and an increased methylation at non-CG sites (Fig 1).

### Progressive reduction of CG methylation in the self-pollinated *ddm1* lines

**Fig 2:**
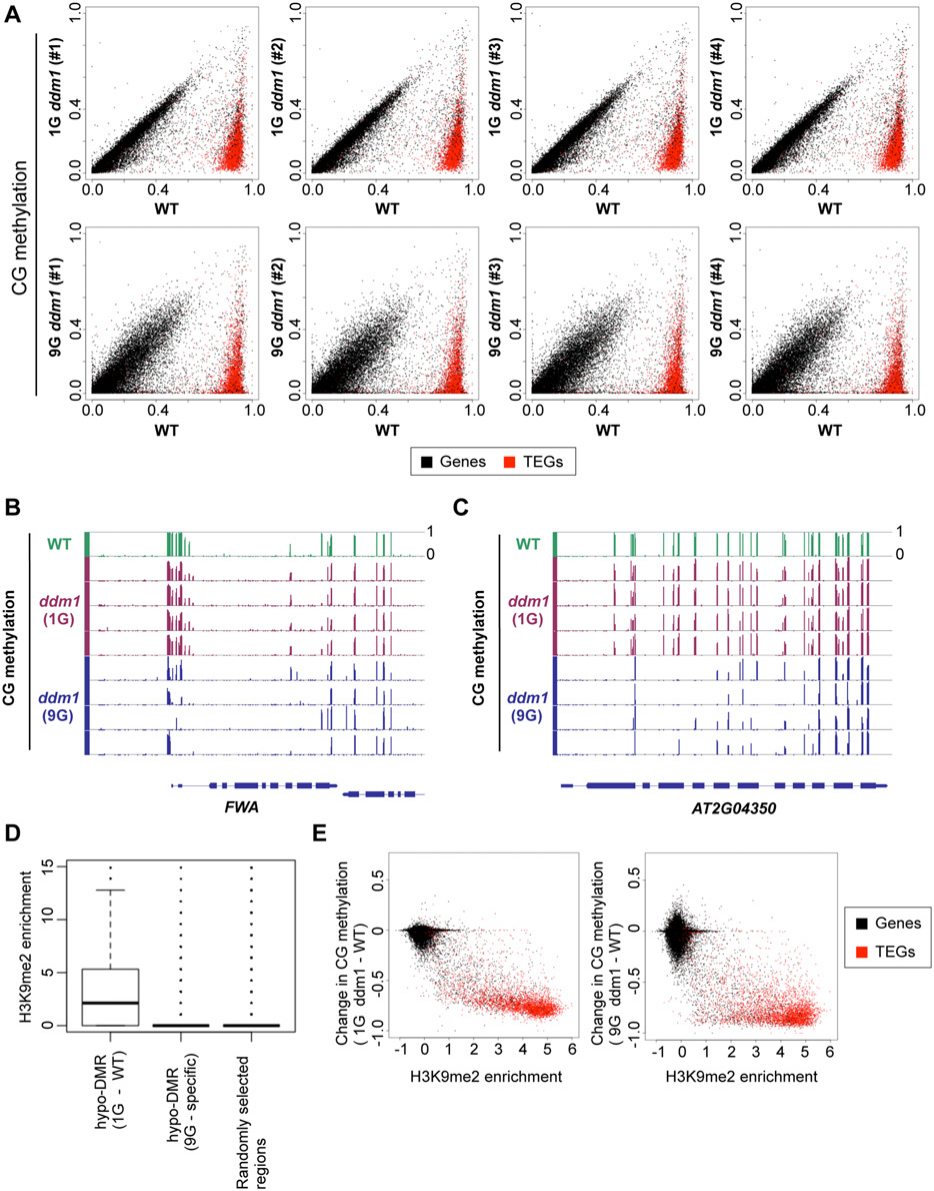
Change of CG methylation during self-pollination of *ddm1* mutants. (A) CG methylation level compared for each transcription unit. Each dot represents the DNA methylation level in a gene (black dot) or a transposable element gene (TEG, red dot). The top half shows effects in four different 1G *ddm1* plants, while the bottom half shows effects in four different 9G *ddm1* plants. Each of the 9G plants was originated from independent self-pollinations. Comparison of the 9G *ddm1* plants to independently self-pollinated 9G *DDM1* plants (S1 Fig) is shown in S3 Fig. “WT” is a *DDM1/DDM1* plant segregating as a sibling of the 1G *ddm1/ddm1* plants. (B, C) Genome browser views of loci with CG methylation reduced in 9G *ddm1* using the Integrated Genome Browser [74]. *FWA* locus (B) and *AT2G04350* locus (C) are shown. The *FWA* gene has dense CG methylation around the 5’ end, which is lost during self-pollination of the *ddm1* mutant. (D) H3K9me2 level of differently hypo-methylated regions (hypo-DMRs) in CG context. Left (1G - WT): Distribution of 119,883 DMRs between WT and 1G *ddm1* mutant. Center (9G - specific): Distribution of 25,861 DMRs between WT and 9G *ddm1*, excluding DMRs between WT and 1G *ddm1*. Distribution of 100,000 randomly chosen 100 bp regions is also shown as a control (right). H3K9me2 level of wild type is shown by reads per million (RPM) in ChIP-seq data obtained from GEO (GSE28398 [72]). (E) Change in CG methylation level in 1G *ddm1* (left) and 9G *ddm1* (right) from wild type, plotted against enrichment of H3K9me2 in wild type (data from Inagaki et al. 2010).

Although the *ddm1* mutation immediately induces a drastic loss of DNA methylation in repeats, further reduction of methylation in later generations has been reported for a few CG sites [30]. Our genome-wide analysis revealed that many loci behave in a similar manner (Fig 2A). The progressive reduction of DNA methylation can have significant phenotypic effects; for example, the promoter of the imprinted gene *FWA* remains methylated in the 1G *ddm1* but the methylation is lost stochastically in 9G *ddm1* (Fig 2B), generating heritable epialleles that cause late-flowering phenotype [31,36,38]. The progressive reduction is seen genome-wide for both genes and TEs (Fig 1A and 1D).

To compare the features of the regions hypomethylated immediately or gradually, we defined differentially methylated regions (DMRs; details in Materials and Methods). The regions *ddm1* affects immediately (1G-WT DMRs) were enriched in dimethylation of histone H3 lysine 9 (H3K9me2) (Fig 2D left and 2E). H3K9me2 is a mark of silent heterochromatin, and these results are consistent with previous reports [10,26]. In marked contrast, however, regions affected slowly (9G-specific DMRs) have much lower level of H3K9me2 in wild type (Fig 2D middle). *DDM1* gene function is necessary for CG methylation in heterochromatin, but in the long-term *DDM1* also has significant effects on CG methylation in less heterochromatic regions including gene bodies (Fig 2C).

### Accumulation of non-CG methylation in *ddm1* lines after propagation by self-pollination

**Fig 3:**
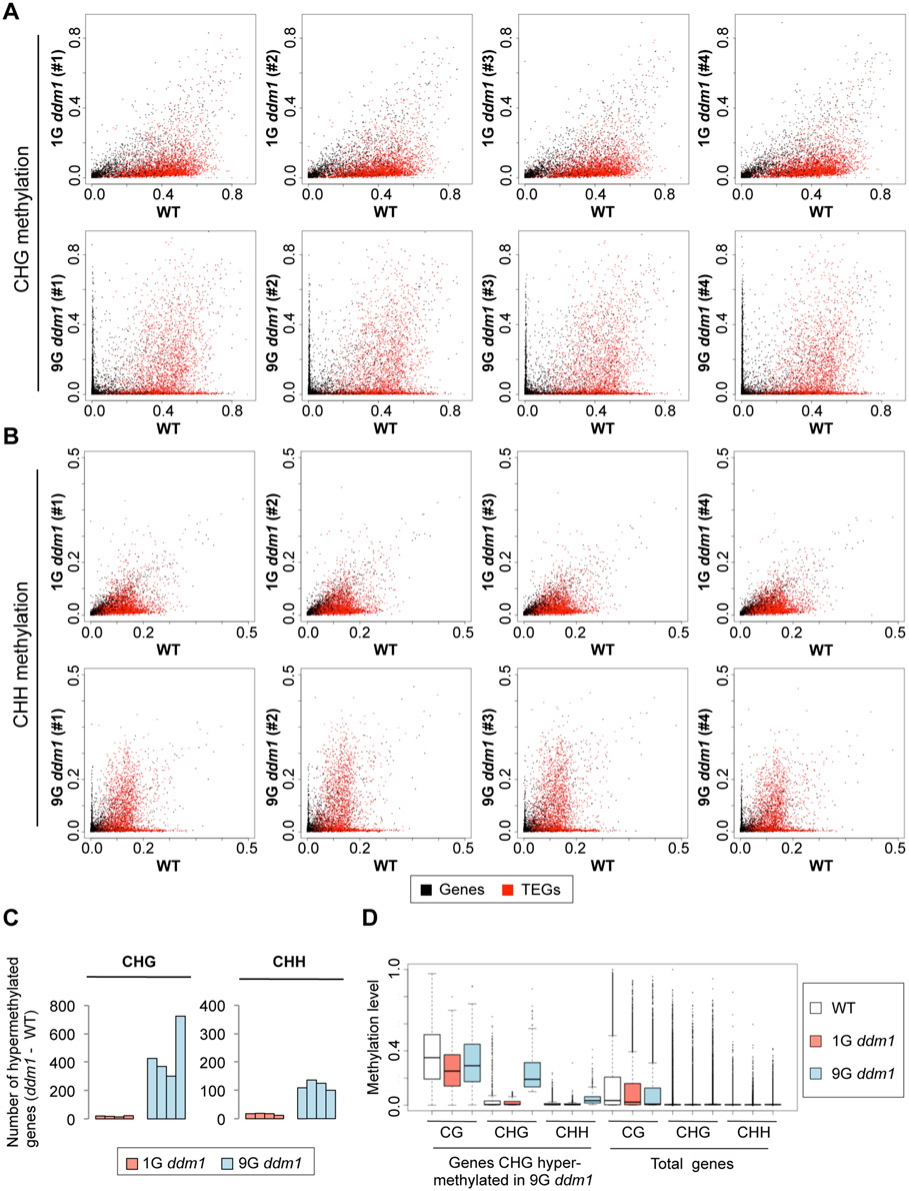
Change of non-CG methylation during self-pollination of *ddm1* mutants. (A, B) Effects of 1G and 9G *ddm1* mutation on CHG methylation (A) and CHH methylation (B). The format is as shown for CG sites in Fig 2A. Comparison of the 9G *ddm1* plants to independently self-pollinated 9G *DDM1* plants is shown in S3 Fig. (C) The number of genes that gained non-CG methylation in *ddm1* mutant (methylation level < 0.1 in WT and ≥ 0.1 in *ddm1*). Results for the four 1G and four 9G of *ddm1* mutants are shown for CHG and CHH sites. (D) Coordinated hypermethylation of CG, CHG and CHH sites. “Genes CHG-hypermethylated in 9G *ddm1*” are those with methylation level < 0.1 in 1G *ddm1* and ≥ 0.1 in 9G *ddm1*. DNA methylation levels for three contexts are shown for WT, 1G *ddm1*, and 9G *ddm1*. On the right, total genes are shown as controls. Although CHG hypermethylated genes tend to have more CG methylation in wild type, the body methylation is not an absolute requirement; even genes without CG methylation occasionally non-CG hypermethylated in 9G *ddm1* (S4 Fig). Pattern of CG methylation change from 1G *ddm1* to 9G *ddm1* is further characterized in S5 Fig.

**Fig 4:**
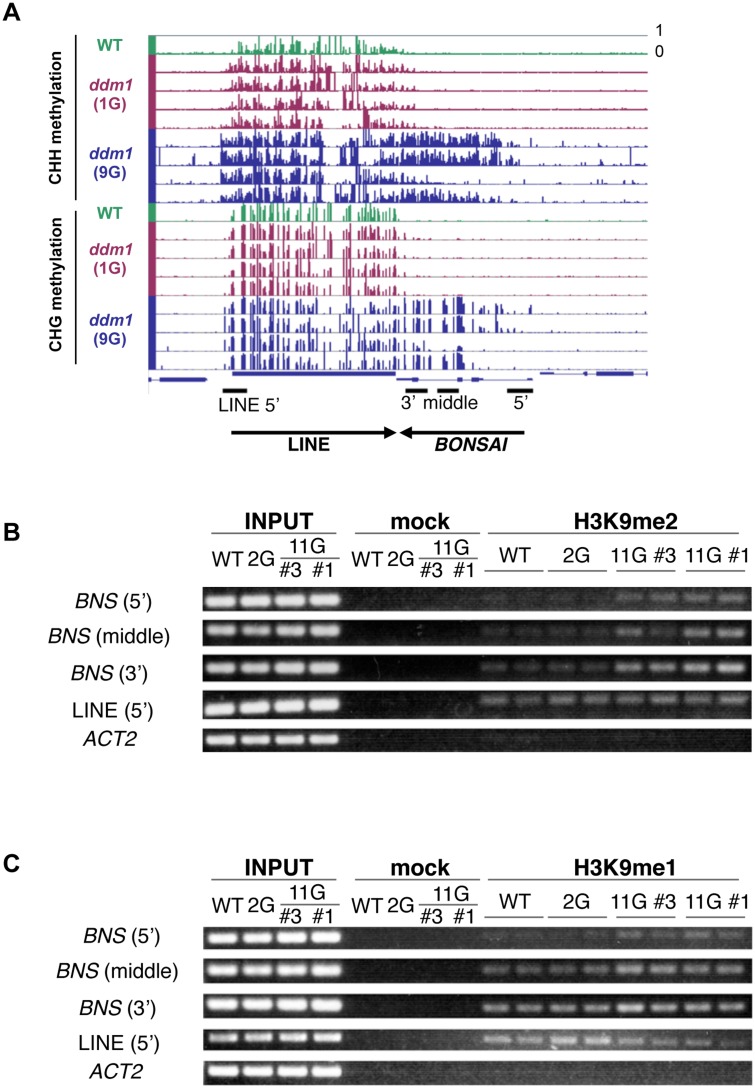
*BONSAI* hypermethylation in self-pollinated *ddm1* mutants is associated with H3K9 methylation. (A) Genome browser views of CHH and CHG methylation in *AT1G73177* (*BONSAI*) locus. (B) H2K9me detected by chromatin immunoprecipitation (IP). “input” is the sample before IP; “mock” denotes samples after IP procedure without antibody. H3mK9me1 and H3K9me2 are samples after IP with the respective antibodies. Amplified regions around the *BONSAI* locus are indicated in (A). LINE and *ACT2* are used as positive and negative controls, respectively. 11G plants are the *ddm1* mutants in the 11th generation. 11G #1 and 11G #3 samples are prepared from progenies of direct sibling of 9G *ddm1* #1 and #3 plants (shown in A), respectively. Results for other loci are shown in S6 and S7 Figs. Although the *BONSAI* locus accumulated both CHG and CHH methylation, some of the CHG hypermethylated loci have less CHH methylation than others (S6A Fig). In our preliminary analyses, H3K9me1 is more prevalent in those loci than H3K9me2 (S6 and S7 Figs).

**Fig 5:**
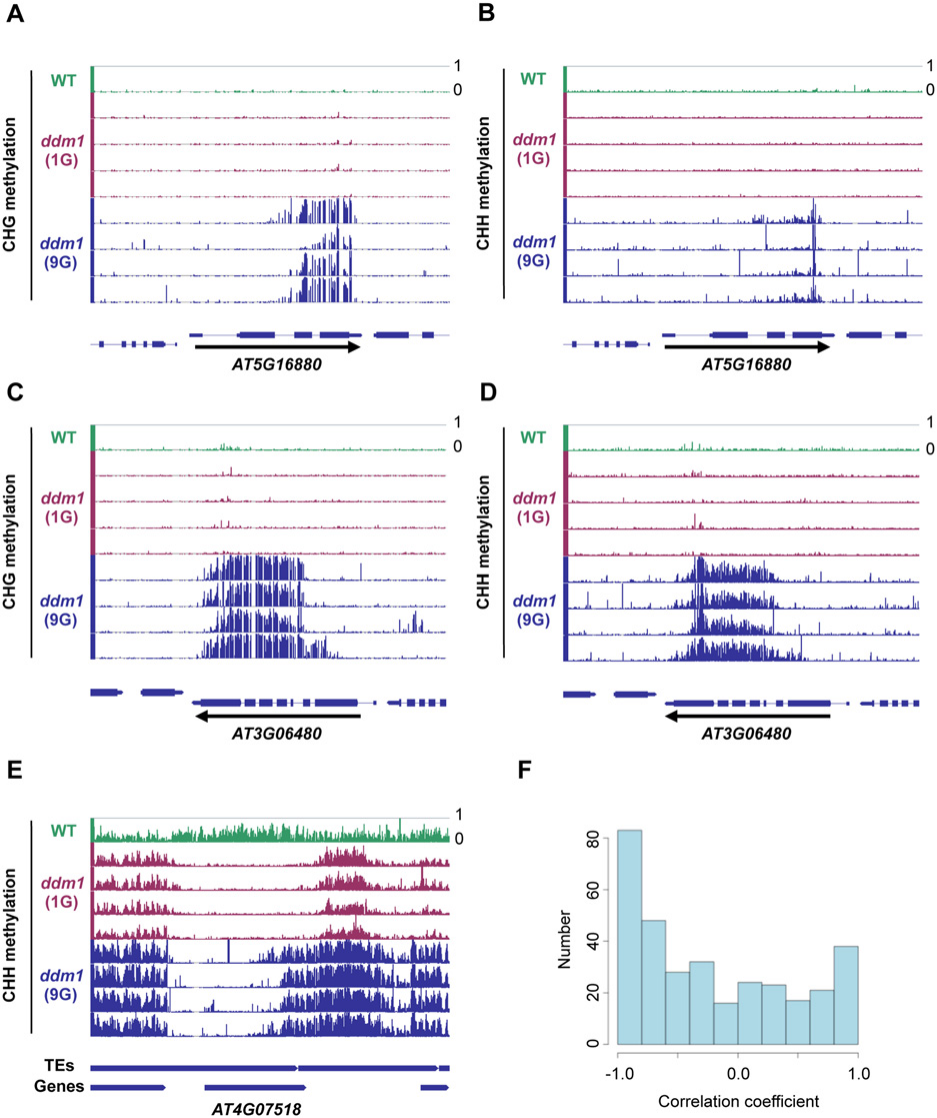
Spread of non-CG methylation in self-pollinated *ddm1* mutants. (A-E) Genome browser views of loci with non-CG methylation in the 9G *ddm1* plants. *AT5G16880* (A-B), *AT3G06480* (C-D), and *AT4G07518* loci (E) are shown for CHG (A, C) and CHH (B, D, E) contexts. Direction of transcription is shown by an arrow in A-D. (F) Histogram of correlation coefficient between the CHG methylation level and the relative centroid position of CHG methylation within the DMR. The centroid position was determined by averaging relative position of the methylated cytosine weighed with the methylation level for each residue. The coefficient was calculated among the four 9G *ddm1* plants in each conDMR for CHG methylation between 9G and 1G *ddm1* (details in Materials and Methods section) overlapping with genes. The coefficient becomes negative when the centroid moves to the 5’ regions as the average level of CHG methylation in the conDMR increase. A large proportion of the contiguous DMRs with the coefficient near -1 reflects spread of CHG methylation from 3’ to 5’ regions as the CHG methylation levels increase.

More counter-intuitively, our genome-wide analysis revealed a large number of genes and TEs ectopically hypermethylated at non-CG sites in the self-pollinated *ddm1* lines (Figs 3A, 3B, 4A and 5A–5E). The regions CHG hypermethylated also showed hypermethylation at CHH sites (Figs 3D, 5A–5D, and S6A Fig). In addition, although genic CG methylation tends to decrease progressively from 1G to 9G on average (Figs 1 and 2), non-CG hypermethylated regions show an increase in CG methylation (Fig 3D). The CG and non-CG hypermethylation was found reproducibly at specific loci (S8 Fig). The affected loci include *BONSAI* and other sequences we have reported previously [37,39], but the majority of the affected loci could only be detected by whole-genome bisulfite sequencing (WGBS), because that can detect increased non-CG methylation with high sensitivity even at loci already CG methylated. In addition to genes, a large number of TEs showed increase in non-CG methylation (Figs 3A,3B, 4E, and S9–S11 Figs).

A very unexpected feature revealed by WGBS is that non-CG hypermethylation of genes is almost undetectable in the first generation of *ddm1* but is specifically and reproducibly seen in the repeatedly self-pollinated *ddm1* lines. In Fig 3A and 3B, many black dots can be seen along the vertical axis in the panels for 9G but not for 1G. The non-CG hypermethylation of genes is not a simple extension of the effect seen in the first generation. This feature can only be detected in later generations (Fig 3C). In order to further understand the transgenerational dynamics, we examined four independently self-pollinated 2G *ddm1* plants. If the hypermethylation proceeds equally at each self-pollination, the increase from 1G to 2G would be 1/8 or more of the increase from 1G to 9G, provided that the methylation level should saturate at specific level (the methylation level can not exceed 100%). Interestingly, although hypermethylation proceeded in 2G, the difference between 1G and 2G was much less than 1/8 of that between 1G and 9G, suggesting that the increase is slow initially but accelerated in later generations (S12 and S13 Figs).

### Spread of H3K9me and non-CG methylation in *ddm1* mutants

How is this non-CG hypermethylation induced? Our genome-wide bisulfite analyses revealed that the genes non-CG hypermethylated in the self-pollinated *ddm1* tend to have low levels of non-CG methylation already in wild type plants (Fig 3D), suggesting that preexisting small heterochromatin domains may function as seed for further heterochromatin formation. Interestingly, distribution of H3K9me2 around the DMR is asymmetric; it is enriched in the 3’ of the DMRs (S14 Fig). We have previously shown that the *BONSAI* gene is flanked by insertion of a heterochromatic LINE in the 3’ region [37] (Fig 4A and S13A Fig). The *BONSAI* hypermethylation in *ddm1* is induced in a strain with the LINE insertion but not found in a strain without the LINE insertion [37]. The DNA methylation spreads from the 3’ LINE to the *BONSAI* region during repeated self-pollination of *ddm1* mutants [37]. Spread of non-CG methylation from 3’ to 5’ regions was also noted at other loci (Fig 5A–5D). When the methylation level differs among the four 9G *ddm1* plants, plants with stronger signals tended to show relative centroid positions more upstream than plants with weaker signals, suggesting that the signal spreads from 3’ to 5’ (Fig 5F). These observations suggest that common mechanisms may operate in *BONSAI* and many, even if not all, affected loci.

We have previously shown that the de novo non-CG methylation in the self-pollinated *ddm1* does not require components of the RdDM machinery, such as RDR2, DCL3, and DRM2 [39]. On the other hand, the non-CG methylase CMT3 and H3K9 methylase KYP are necessary for the de novo methylation, suggesting that the ectopic methylation occurs by mechanisms mediated by the heterochromatin marks H3K9me and non-CG methylation [39]. Indeed, the non-CG hypermethylation at the *BONSAI* locus is associated with ectopic H3K9me (Fig 4B).

The self-reinforcing loop of non-CG methylase and H3K9 methylase activities could be the basis for the acceleration of hypermethylation as the generation proceeds (S13B Fig). As the two processes enhance each other, the positive feedback would accelerate the spread of the heterochromatin in later generations [12, 13].

### Properties of loci hypermethylated in the self-pollinated *ddm1*

**Fig 6:**
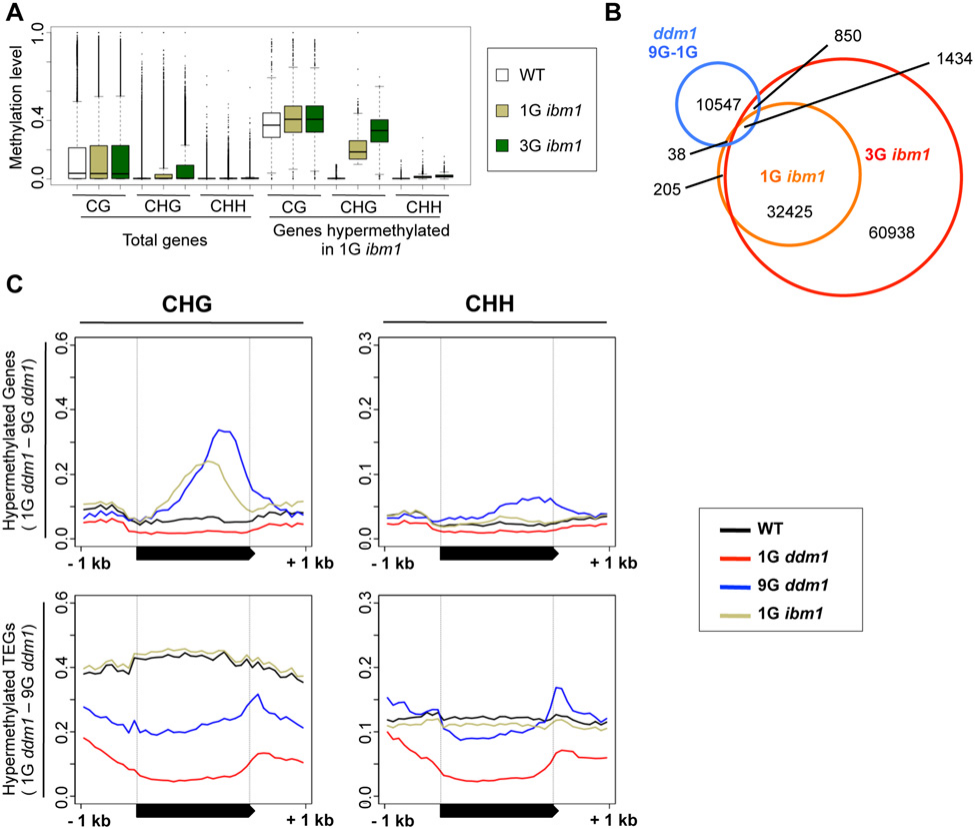
Hypermethylated regions in *ddm1* and *ibm1* mutants. (A) Increase of CHG methylation in 1G and 3G *ibm1* mutants. Genes hypermethylated in 1G *ibm1* (CHG methylation level < 0.1 in WT and ≥ 0.1 in 1G *ibm1*) are shown (right) with total genes (left). Profiles for multiple 1G and 3G *ibm1* mutant plants are shown in S15 Fig. (B) Comparison of regions CHG hypermethylated in *ibm1* and 9G *ddm1*. DMRs between 9G and 1G *ddm1* (blue), between 1G *ibm1* and WT (orange), and between 3G *ibm1* and WT (red) are shown. Heat map of CHG methylation for these DMRs are shown in S16B Fig. (C) DNA methylation profile for the genes CHG hyper-methylated in 9G *ddm1* (shown in Fig 3D). The top and bottom half represent genes and TEGs, respectively. In these regions, CHH methylation also increased in 9G *ddm1*.

Increased non-CG methylation has been reported in mutants of the CG methyltransferase gene *MET1* [40–42], which results at least in part from a reduction of full-length *IBM1* transcript [43]. The *IBM1* gene encodes a demethylase for histone H3K9; and mutation in this gene induces accumulation of H3K9me2 and non-CG methylation in gene bodies. Interestingly, developmental phenotypes of the *ibm1* mutation also become progressively stronger during self-pollinations [15]. We compared the regions of non-CG hypermethylation in the *ibm1* and self-pollinated *ddm1*. Although an overlap can be detected, the majority of the DMRs in *ddm1* mutants before and after the self-pollinations were distinct from the DMRs of *ibm1* mutants (Fig 6B and S16 Fig). Just as progressive loss of CG methylation in the *ddm1* mutant, *ibm1* mutant shows progressive accumulation of non-CG methylation in later generations (Fig 6A, S15 and S16 Figs). This is consistent with a recent report [44] and likely accounts for the progressive developmental defects in the *ibm1* mutant.

We examined DNA methylation patterns of the genes and TEs hypermethylated in the self-pollinated *ddm1* lines (Fig 6C). Compared to the *ibm1* mutant, the peak in the *ddm1* was shifted toward 3’ end. Interestingly, the shift of the peak in the hypermethylation was also found for CG methylation (S5D Fig). Although CG methylation of gene body in wild type peaks around the center (S5C Fig), increase of genic CG methylation in 9G *ddm1* was not proportional to the methylation of wild type; instead, the increase of CG methylation was shifted toward 3’ regions (S5D Fig). Together with the observation that CHG-hypermethylated genes tend to show CG-hypermethylation (Fig 3D), these results suggest a link between the ectopic CG methylation and non-CG methylation, as we discussed previously [39].

The bias of the hypermethylation signal toward the 3’ region in 9G *ddm1* is especially evident in the hypermethylated TEs; the peak was often located outside of the transcription unit for both CHG and CHH methylations (Fig 6C, bottom half). When different families of TEs are compared, the peak in the downstream region was especially evident in the GYPSY-like LTR retrotransposons (S10 Fig). Generally, these TEs lost DNA methylation in 1G *ddm1*, but regained methylation during the self-pollinations (S5A and S9–S11 Figs).

### The hypomethylated chromosomes from a *ddm1* mutant could induce hypermethylation in trans even in a *DDM1* wild type background

The *ddm1* mutation can induce increased DNA methylation at hundreds of genes and TEs. The hypermethylation can be a direct consequence of impaired *DDM1* function, or alternatively, an indirect effect of disruption of heterochromatin in the mutants. To test these possibilities, we examined the effect of chromosomes introduced from *ddm1* into wild type *DDM1* background. Chromosomes losing DNA methylation in the *ddm1* mutants remain unmethylated even after introduction into wild type *DDM1* background [25,45]. We examined DNA methylation data of epigenetic recombinant inbred lines (epiRILs) [46]. In the epiRILs, a *ddm1* mutant plant was crossed to wild type plant twice to segregate *DDM1/DDM1* lines with around one quarter of chromosome segments derived from *ddm1*. Although remethylation can be induced in regions associated with small RNA, hundreds of DMRs remain unmethylated in the wild type *DDM1* background [46,47]. Each of these segregating lines have been self-pollinated seven times, which makes most of the genomic regions fixed in *ddm1*-derived haplotype or wild-type derived haplotype [46].

**Fig 7:**
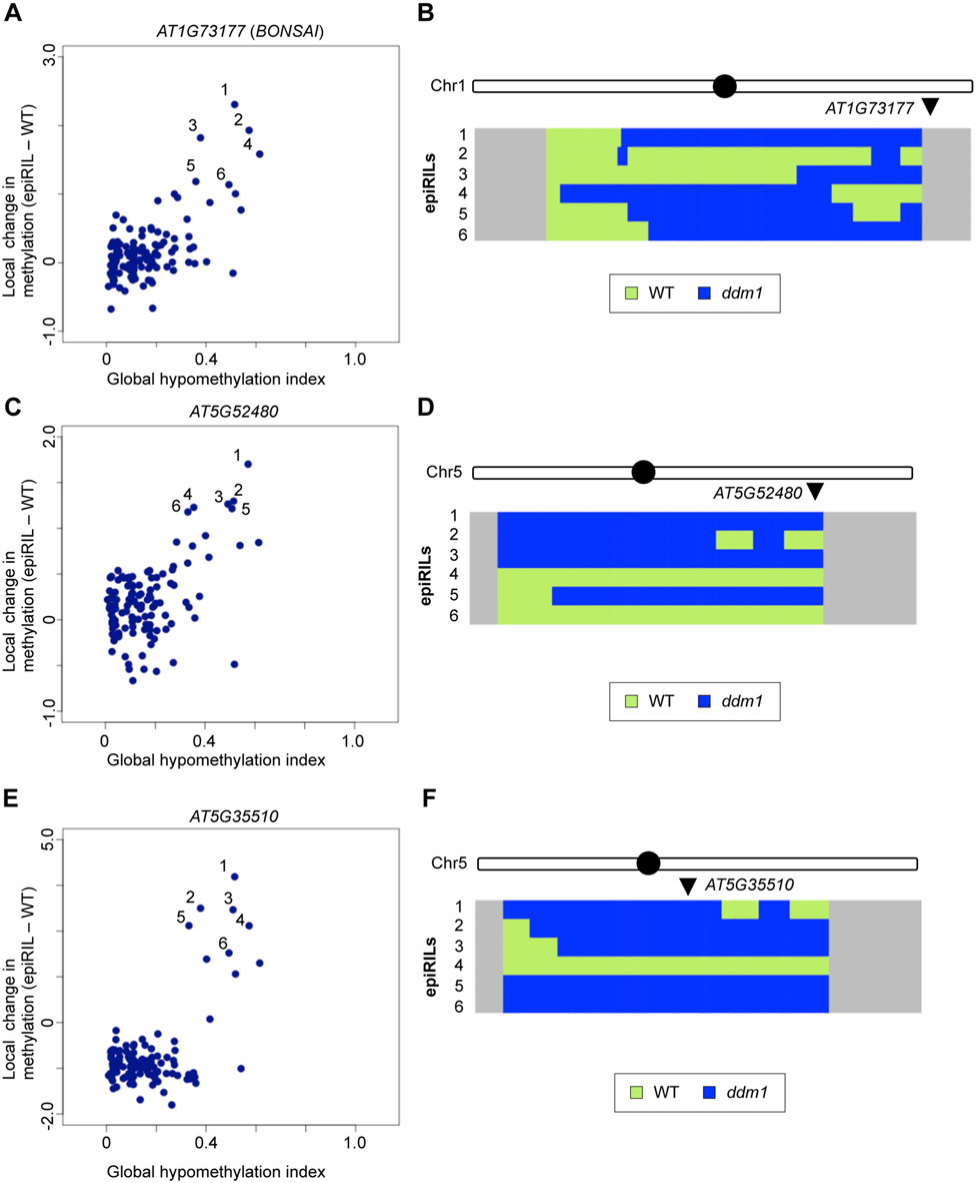
Effects of disrupted heterochromatin in the *DDM1* wild type background examined by IP. (A, C, E) Changes in local DNA methylation plotted against the global level of DNA hypomethylation in 123 epigenetic recombinant inbred lines (epiRILs). Each dot represents the value for one line. Three loci, *AT1G73177* (*BONSAI*) (A), *AT5G52480* (C), and *AT5G35510* (E) are shown. Results with four other loci are shown in S17 Fig, with values for the F_0_ *ddm1* and WT parents. (B, D, F) WT (light green) / *ddm1* (dark blue) haplotype for epiRILs that showed increase of cytosine methylation for each locus (numbered 1–6 for each locus, the line names can be different among the panels). In each panel, the chromosome including the target locus (arrowhead) is shown. Haplotypes of all five chromosomes are shown in S18–S23 Figs. The filled circles indicate centromere positions. The haplotypes are predicted from stably hypomethylated markers [46]. The regions not covered by any markers are indicated in gray. Names of epiRILs numbered 1–6 in each panel are in Materials and Methods. Data of epiRILs were obtained from GEO (GSE37284 [46]).

We examined if the loci exhibiting hypermethylation in the self-pollinated *ddm1* lines also showed hypermethylation in some of the epiRILs. We utilized DNA methylation data for the 123 epiRILs, which are based on immunoprecipitation (IP) of genomic DNA by anti-methylcytosine antibody. As the context of methylation cannot be distinguished, we examined seven loci that show increased methylation in 9G *ddm1* but a relatively low level of methylation at CG sites in wild-type. In six out of the seven loci examined, we could detect hypermethylation in multiple epiRILs, suggesting that the hypermethylation can be induced or maintained in the *DDM1* background (Figs 7A, 7C, 7E and S17 Fig). In all of them, the hypermethylation showed a strong positive correlation with the amount of disrupted heterochromatin in each of these lines (Fig 7, S17 Fig and S1 Table), suggesting that the hypermethylation was induced or maintained in the background of disrupted heterochromatin in other genomic regions.

The hypermethylation could be induced de novo or alternatively maintained from the parental *ddm1*. The parental *ddm1* plant originally used for making epiRILs was already self-pollinated three times (4G) and that plant also show low level of ectopic methylation at some loci (S17 Fig), which may have the potential to be maintained in *DDM1* background [37]. Very importantly, however, the hypermethylation was found even in chromosome segments originated from wild type *DDM1* (Figs 7B, 7D, 7F and S18–S23 Figs), demonstrating that the hypermethylation could be induced de novo after the initial crosses and subsequent repeated self-pollinations in the background of functional *DDM1*.

**Fig 8:**
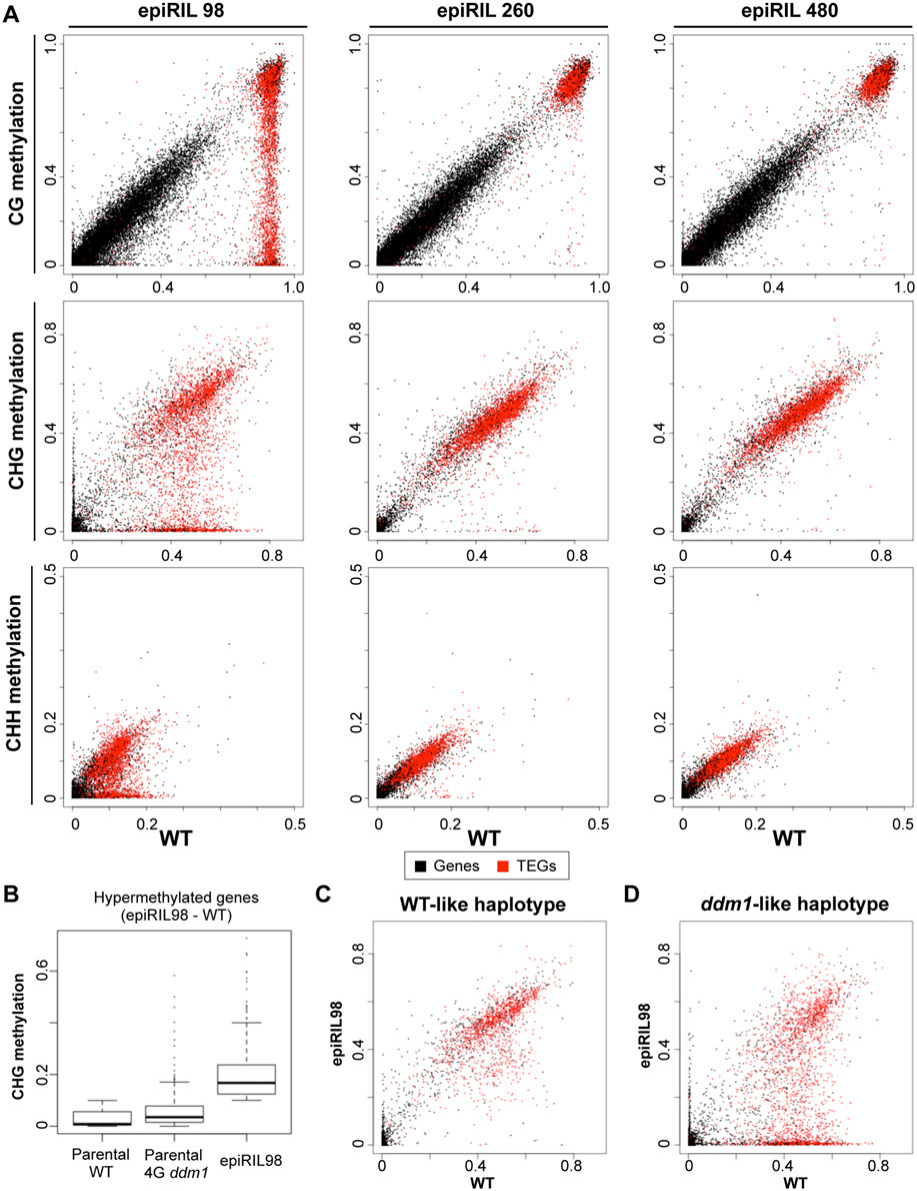
Effects of disrupted heterochromatin in the *DDM1* wild type background examined at single base resolution. (A) Methylation level was compared for each transcription unit in CG, CHG, and CHH contexts. The format is as shown in Fig 2A. A globally hypomethylated epiRIL (epiRIL98: plant #3 in Fig 7A and 7B and plant #2 in Fig 7E and 7F) and two epiRILs with lower level of hypomethylation (epiRIL260 and epiRIL480) are shown. Global hypomethylation indexes of epiRIL98, epiRIL260, and epiRIL480 are 0.38, 0.04, and 0.09, respectively. “WT” data are from the parental wild-type Col plant used to generate the epiRILs. (B) CHG methylation levels in the genes that were not methylated in WT but methylated in epiRIL98 (methylation level < 0.1 in WT and ≥ 0.1 in epiRIL98: n = 232). For these transcription units, distributions of the methylation levels were compared among the parental WT, the parental 4G *ddm1* plant, and the epiRIL98. (C-D) Ectopic CHG methylation in epiRIL98 compared to wild type. Each gene was assigned to the inferred haplotypes in epiRIL98: WT-like (C) or *ddm1*-like (D). The ectopic methylation could be detected in genes of the WT-like haplotype. Examples of such genes are shown in S25 Fig.

In order to confirm and extend this observation, we used WGBS for an epiRIL with genome-wide reduction of heterochromatic DNA methylation. The epiRIL98, which contains large amount of chromosomes with reduced DNA methylation, showed CHG hypermethylation in many genes (Fig 8A), which include *BONSAI* gene (S24A Fig) and genes with body methylation (S24B–S24C Fig). In the CHG hypermethylated genes, the CHG methylation level was generally much higher than that of the parental 4G *ddm1* plant (Fig 8B), suggesting that the hypermethylation was amplified or induced de novo in the background of functional *DDM1*. A large number of CHG hypermethylated genes were found in chromosome regions of wild type haplotype (Fig 8C and S25 Fig), again suggesting that they can be induced de novo. In control epiRILs with much lower levels of disrupted chromatin, the hypermethylation was undetectable, confirming that the disrupted heterochromatin was responsible (Fig 8A). Taken together, these results indicate that the hypermethylation can be induced de novo by trans-acting effects of disrupted heterochromatin.

## Discussion

### Local spread of heterochromatin by positive feedback loop

Here we report short- and long-term effects of the *ddm1* mutation. The mutation immediately induces a drastic loss of DNA methylation in heterochromatic regions in the first generation when it becomes homozygous. In later generations, the *ddm1* mutation reproducibly induces ectopic accumulation of DNA methylation in hundreds of genes and TEs. This work and previous work [39] suggest that the ectopic methylation occurs by spread of heterochromatin marks mediated by the non-CG methylase CMT3 and H3K9 methylase KYP. Interestingly, this effect was slow in the initial generations but accelerated in later generations, suggesting strong positive cooperativity for the heterochromatin accumulation. That could be explained by the self-reinforcing positive feedback of H3K9me and non-CG methylation [12, 13].

### Global negative feedback for heterochromatin redistribution

**Fig 9:**
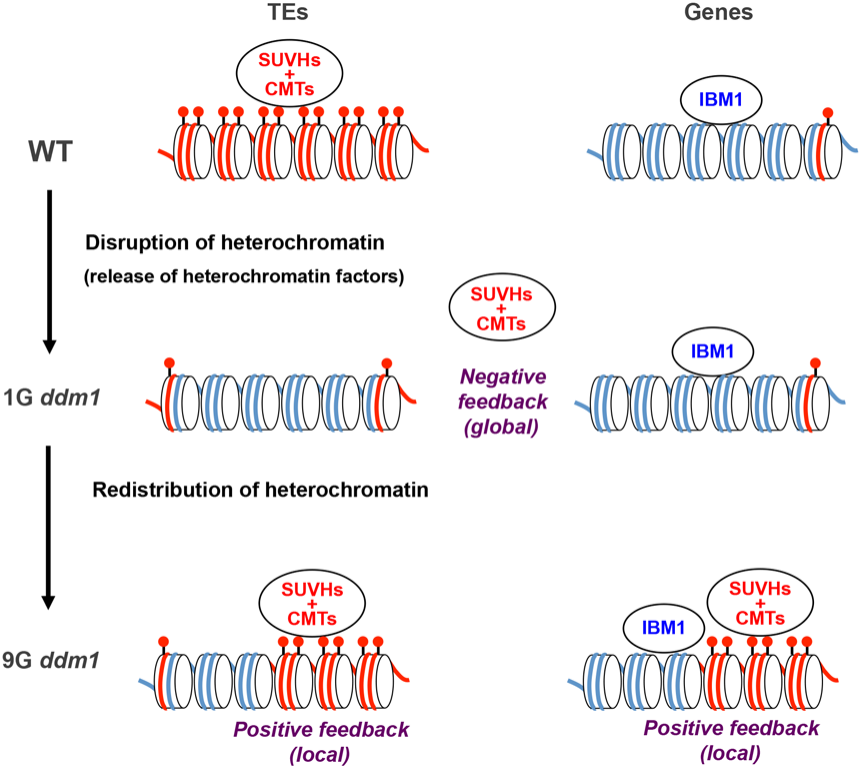
A model for the transgenerational heterochromatin redistribution. The cylinder indicates a nucleosome. Red dots above the nucleosome indicate methylation of H3K9. Red and blue lines indicate DNA with and without non-CG methylation, respectively. The CMTs are non-CG methylases, such as CMT3 and CMT2 [10,11]. SUVHs are H3K9 methylases, such as SUVH4/KYP, SUVH5 and SUVH6 [75]. In both WT and *ddm1* mutant plants, the histone demethylase IBM1 removes H3K9me from transcribed genes.

In addition to the local positive feedback, global negative feedback seems important for the DNA methylation dynamics. The ectopic DNA methylation seems to reflect negative feedback of disrupted heterochromatin in other genomic regions, because the ectopic methylation could also be induced in *DDM1* wild type background when the genome contains large amount of chromosomal segments with disrupted heterochromatin (Figs 7 and 8). How does the negative feedback work? One possible explanation is that disruption of heterochromatin in the *ddm1* mutant results in release of heterochromatin-forming factors such as CMTs and H3K9 methylases, which then become available in other regions. As these factors are normally recruited to heterochromatin, disruption of a large proportion of heterochromatin in the genome would result in increased level of these factors in released conditions, which would induce spread of heterochromatin into normally euchromatic regions and its amplification by the self-reinforcing loop of H3K9me and non-CG methylation (Fig 9).

In the model we proposed, global reduction of heterochromatin induces ectopic non-CG methylation (Fig 9). That would account for the correlation between the global reduction of methylation and ectopic methylation in epiRILs (Fig 7A, 7C, 7E and S17 Fig). An alternative mechanism would be that *ddm1* induces change in a specific locus, such as transcriptional de-repression or repression of a specific gene, and the change is inherited in the *DDM1* wild type background and induces the ectopic methylation. For example, *ROS1* gene expression is reduced in mutants with reduced DNA methylaiton [48], which would lead to hypermethylation at specific loci. However, although *ROS1* gene expression is reduced in *ddm1*, it is expressed almost normally in epiRIL98, which show strong non-CG hypermethylation (S26A Fig). In addition, DMRs hypermethylated in 9G *ddm1* and *ros1*-*dml2-dml3* triple mutant do not overlap much, further suggesting that the hypermethylation in 9G *ddm1* is not due to reduced *ROS1* expression (S26B Fig). More generally, we could not find a locus consistently derived from *ddm1* parent in all of the plants showing the high level of ectopic hypermethylation in the six loci (S18–S23 Figs). Although we cannot exclude the possibility that two or more specific loci redundantly mediate the ectopic methylation, a more parsimonious explanation derived from available data would be that the trans-interaction is mediated by global homeostasis.

The de novo methylation in the epiRILs might also be related to mechanisms such as paramutation [49,50], or transchromosomal methylation (TCM) [51]. In these phenomena, methylated sequences induce methylation in related sequences. However, the ectopic hypermethylation in the epiRILs is generally much higher than that of the parental *ddm1* (Fig 8B), suggesting that even if paramutation-like or TCM-like mechanisms are involved, the effect should be much amplified during self-pollinations of epiRILs; and the degree of the amplification correlates with global disruption of heterochromatin (Fig 7 and S17 Fig), which is due to the *ddm1*-derived chromosomes.

This trans-acting negative feedback could also be understood as a hypersensitive reaction to the challenge by active and proliferating TEs. Our genome-wide analyses revealed that many of the TEs can be targets of the negative feedback (Fig 3A and 3B and S9–S11 Figs). Active TEs often keep parts of heterochromatin, which can function as seeds of the self-reinforcing heterochromatin formation.

An increase in non-CG methylation is also seen in mutants of the histone demethylase gene *IBM1*. However, targets of IBM1 are generally euchromatic and they do not overlap much with regions hypermethylated in the self-pollinated *ddm1* lines (Fig 6B and S16 Fig). An increase in non-CG methylation is also found in the maintenance CG methylase gene *MET1* [40–42]. As a mechanism for the *met1*-induced increase in non-CG methylation, loss of *IBM1* function is suggested, as *IBM1* transcripts become truncated in the *met1* mutant [43]. On the other hand, it has been reported that the main targets of the *met1*-induced accumulation of H3K9me2 are genes with H3K27me3, another modification for silent chromatin [52]. The negative feedback of heterochromatin marks comparable to that seen in the self-pollinated *ddm1* lines may also operate in *met1* mutants. In our analyses, although regions affected by *met1*, *ibm1*, and self-pollinated *ddm1* all differ, significant overlaps are noted (S27 Fig). For these mutants, the local triggers for heterochromatin accumulation appear to be distinct, despite the possible overlap in the downstream mechanisms, including the self-reinforcing loop of non-CG methylation and H3K9me.

### Perspective

Heterochromatin homeostasis mechanisms analogous to those we have uncovered in Arabidopsis may also be operating in other eukaryotes. Mice with a disruption of its *DDM1* homolog *Lsh* show global reduction of genomic DNA methylation, but interestingly it is also associated with increased DNA methylation at specific regions [29]. In human cancer, hypomethylation of repeats and TEs is often associated with local hypermethylation of genes, such as tumor suppressor genes [53,54]. In Drosophila, an increase in the amount of heterochromatic Y chromosome can results in a release of silencing at multiple loci in trans [55], suggesting a negative feedback similar to that discussed here. Furthermore, Drosophila modifiers of position effect variegation often function in dosage-dependent manners [56,57], consistent with the pathway proposed in Fig 9. Positive feedback loops would stabilize and enhance silent and active states [12,13,21,58], but they carry the risk of going out of control to excess. A global negative feedback mechanism, together with the local positive feedback, would ensure a robust and balanced chromatin differentiation within the genome, as has been discussed for pattern formation during development [59,60].

In the context of evolution in plants, a large variation in the amount of repetitive sequences is often noted between related species or even within a species [61–63]. On such occasions, fine-tuning of the amount of trans-acting heterochromatin factors would be especially important, as an imbalance would not only immediately affect gene expression level but also influence the epigenotype in a transgenerational manner.

## Materials and Methods

### Plant materials and annotations

Isolation of the *ddm1-1* and *ibm1-4* mutants has been described previously [15,25]. Self-pollination of *ddm1* lines was described previously [30]. In order to remove heritable effects of the *ddm1* mutation, the original *ddm1* mutant was backcrossed six times in the heterozygous state. The heterozygous plants were propagated by self-pollination. 1G *ddm1* mutant plants were selected from self-pollinated progeny of the heterozygote. 9G *ddm1* plants were generated by independently self-pollinating different *ddm1* segregants eight times (S1 Fig). Generation of epiRILs has been described previously [64].

The annotations of genes and TEs are based on The Arabidopsis Information Resource (http://www.arabidopsis.org/). TAIR8 was used for analyzing ChIP chip data (Fig 2E), TEG (TE gene) data, and epiRILs data. TAIR10 was used for other analyses. The details of the annotation of TEGs were described in a document in TAIR web (ftp://ftp.arabidopsis.org/home/tair/Genes/TAIR8_genome_release/Readme-transposons).

### DNA methylation analyses

For the 1G and 9G *ddm1* plants and their controls, genomic DNA was isolated from rosette leaves using the Illustra Nucleon Phytopure genomic DNA extraction kit, and genome-wide bisulfite sequencing was performed as described previously [65]. Raw sequence data were deposited in the DDBJ (DNA Data Bank of Japan) Sequence Read Archive (DRA; accession nos. DRA002545, DRA002546, DRA002548, DRA002549, DRA002551, DRA002554, DRA002555, DRA003018, DRA003019 and DRA003020). The adaptor sequences were clipped out using the FASTX-toolkit (http://hannonlab.cshl.edu/fastx_toolkit/). Reads were trimmed to 90 nucleotide length (45 nucleotide for the data obtained from GEO—GSE39901) and mapped to reference genomes (Release 10 of the Arabidopsis Information Resources) using the Bowtie alignment algorithm [66] with the following parameters, "-X 500-e 90-l 20-n 1". Only uniquely mapped reads were used. Clonal reads were removed except one with the best quality. Any read with three consecutive methylated CHH sites were eliminated. The level of methylation of cytosine in a genomic region was calculated using the ratio of the number of methylated cytosine to that of total cytosine. For the three epiRILs and two parental lines, whole-genome bisulfite sequencing was described previously [46] and the data are in GEO (GSE62206).

DMRs (differentially methylated regions) were defined by comparing the methylation level of 100-bp windows throughout the genome between two genotypes. The windows with at least 20 sequenced cytosines were used for the comparison. The level of methylation was calculated using the weighed methylation level of each genotype [67]. The windows were selected as DMRs when difference of methylation level was 0.5 or more at CG site or 0.3 or more at CHG sites. For defining contiguous DMR (conDMR), multiple DMRs were merged if they were adjacent to each other or there was only one gap of the 100-bp window. The centroid of cytosine methylation in conDMR was calculated using the relative position within that region weighed by methylation level of each cytosine. In Fig 5F, we used conDMR of 500 bp or longer and overlapping with genes. Each contiguous DMR was aligned according to the orientation of the corresponding gene. The correlation coefficient between the level and the relative centroid position of DNA methylation was calculated among the four 9G *ddm1* plants in each conDMR. To plot DNA methylation patterns over genes or TEGs in *ddm1* mutants, #1 samples of each genotype (Figs 2A, 3A and 3B) in 1G *ddm1* and 9G *ddm1* were used. To draw the heatmap of methylation of cytosine, cluster 3.0 [68] and Java Treeview [69] were used.

### Chromatin IP analysis

15-day-old seedlings were fixed with formaldehyde and ChIP was performed as described previously [70], using antibody against H3K9me1 (CMA316) and H3K9me2 (CMA307) [71]. To assure the equal amount of chromatin in each line, input DNA were quantified by quantitative PCR using TaKaRa Dice_Real Time System TP800 and ACT7 primers. Then, input DNA and each sample were diluted according to the estimated input DNA concentrations. Input DNA, mock (without antibody), and ChIP samples were analyzed by PCR. The PCR conditions were as follows: pre-incubation for 2 min at 94°C, 27 cycles at 94°C for 30 sec, 58°C for 20 sec, 72°C for 45 sec and a final extension at 72°C for 4 min. Primers used for the ChIP are listed in S2 Table. In addition to the *BONSAI* locus, we examined six loci with CHG methylation increased more than 0.3 from 1G *ddm1* to 9G *ddm1*. Three of them were selected for relatively high level of ectopic CHH methylation (H1, H2, H3) and three with relatively low CHH methylation (L1, L2, L3). The increase of CHH methylation from 1G *ddm1* to 9G *ddm1* is more than 0.2 for the three H loci, and it is less than 0.02 for the three L loci. The lengths of amplicons for the six loci are between 250 bp and 300 bp.

### Processing ChIP-seq data

ChIP-seq data of various histone modifications [72] in GEO (GSE28398) were used for our analysis. The coordinates were remapped onto TAIR10 annotation using a script in TAIR [73]. Enrichment of histone modification in a DMR was calculated by the density of ChIP-seq reads, and normalized by the mean and the standard deviation of the density of reads in 100,000 windows randomly chosen across the genome.

### Processing MeDIP-chip data of epiRILs

The MeDIP-chip data of 123 epigenetic recombinant inbred lines (epiRILs), *ddm1* and WT are in GEO (GSE37284). The regions that were methylated (M) in WT and unmethylated (U) in *ddm1* were selected as targets of *ddm1* mutation using the values for HMM (hidden Markov model) status (M (methylated) or I (Intermediate) or U (Unmethylated)) [46]. Global hypo-methylation index of an epiRIL was calculated as the genome-wide average of the values for HMM status of probes on the chip (M = 0, I = 0.5, U = 1) in the target regions of *ddm1* mutation. The data of inference of inherited haplotypes were shown in the previous study [46]. Following are names of lines numbered 1–6 in Fig 7 and S18–S23 Figs. (Fig 7A and 7B and S18 Fig) epiRIL208 epiRIL122 epiRIL98 epiRIL232 epiRIL70 epiRIL114; (Fig 7C and 7D and S19 Fig) epiRIL122 epiRIL208 epiRIL114 epiRIL258 epiRIL438 epiRIL508; (Fig 7E and 7F and S20 Fig) epiRIL208 epiRIL98 epiRIL438 epiRIL508 epiRIL122 epiRIL114; (S21 Fig) epiRIL208 epiRIL73 epiRIL71 epiRIL394 epiRIL98 epiRIL438; (S22 Fig) epiRIL508 epiRIL114 epiRIL122 epiRIL438 epiRIL208 epiRIL93; (S23 Fig) epiRIL208 epiRIL114 epiRIL556 epiRIL71 epiRIL244 epiRIL98.

## Supporting Information

S1 Table Strong positive correlation between the global hypomethylation and the local hypermethylation in epiRILs. Pearson correlation coefficients are shown with p-values using the data of 123 epiRILs. Six out of the seven loci examined in S17 Fig showed strong positive correlation.
(XLSX)

S2 Table Primers used for ChIP. (XLSX)

S1 Fig Production of self-pollinated *ddm1* and control *DDM1* lines. Genetic scheme of the production of self-pollinated *ddm1* and control *DDM1* lines. The parental *DDM1/ddm1* (shown as *D/d*) is generated by backcrossing original *ddm1* mutant to wild type six times in the heterozygous state. In the self-pollinated progeny of the heterozygote, multiple *ddm1/ddm1* (*d/d*) and *DDM1/DDM1* (*D/D*) plants were selected and self-pollinated eight times independently to generate 9G *ddm1* and control 9G *DDM1* plants
(TIF)

S2 Fig Chromosome-wide view of DNA methylation profiles in *ddm1* mutant lines before and after self-pollinations. Cytosine methylation levels are shown for the three contexts, CG, CHG and CHH, with the sliding windows of 1Mb.
(TIF)

S3 Fig Change of DNA methylation in 9G *ddm1* plants compared to control 9G *DDM1* plants. Methylation level of cytosine was compared for each transcription unit between 9G *DDM1/DDM1* plants and WT in the CG (A), CHG (B), and CHH (C) contexts. The format is as shown in Fig 2A. Each of the 9G plants was originated from independent self-pollinations (S1 Fig). “WT” is a *DDM1/DDM1* plant segregating as a sibling of the 1G *ddm1/ddm1* plants.
(TIF)

S4 Fig Ectopic non-CG methylation found in loci without CG methylation in WT. Methylation level of cytosine was compared for each transcription unit. (A) Change in CHG methylation between 1G and 9G *ddm1* plotted against CG methylation level in WT. Although many of the body-methylated genes show the ectopic CHG methylation, substantial number of unmethylated genes also showed the ectopic CHG methylation. (B-C) Two examples of genes without CG methylation in WT, but gained CHG methylation in 9G *ddm1*. In these loci, the 9G *ddm1* also showed low level of ectopic CG methylation. The ectopic CG hypermethylation accompanied by non-CG methylation is also found in other loci [39].
(TIF)

S5 Fig Pattern for increase of CG methylation in 9G *ddm1*. Methylation level of cytosine was compared for each transcription unit. (A) Comparison of two changes of CG methylation, (from WT to 1G *ddm1*) and (from 1G *ddm1* to 9G *ddm1*). Regression lines for the data of genes (black) and TEGs (red) were calculated using least square method. The two changes correlate negatively in TEs reflecting that some TEs loose methylation in 1G *ddm1* but regained that in 9G *ddm1*. In contrast, The correlation was positive in genes, suggesting that many genes accumulate CG methylation in 9G *ddm1*, even though they do not loose methylation in 1G *ddm1*. (B) Change in CG methylation from 1G to 9G *ddm1* plotted against CG methylation level in WT. Some genes accumulate CG methylation in 9G, even if they do not have CG methylation in WT. The results are analogous to that in S4A Fig; ectopic CG and non-CG methylation can accumulate in 9G *ddm1* even for genes without CG methylation in wild type. (C) Patterns of CG methylation for the genes CG hypermethylated in 9G *ddm1* (genes defined as “hypermethylated” for at least three lines in S8B Fig) compared among WT, 1G *ddm1* and 9G *ddm1*. (D) The pattern of difference of CG methylation between WT and 9G *ddm1* over the genes hypermethylated in CG context. The peak of the increase was shifted to 3’ region, compared to peak in CG body methylation shown in (C).
(TIF)

S6 Fig H3K9me in the CHG hypermethylated loci. (A) Change in CHG methylation and CHH methylation during self-pollination of *ddm1*. TEGs (red) tend to show more CHH methylation than genes (black) with similar level of CHG methylation. In addition, some genes show more CHH methylation than others with similar level of CHG methylation. (B-C) Genome browser views of CHH and CHG methylation around *AT5G15890* (B) and *AT1G06460* (C) loci. H1 and L1 are regions amplified in (D). H1 and L1 regions were selected for relatively high and low level of ectopic CHH methylation, respectively (details in Materials and Methods section). Both were CHG hypermethylated in 9G *ddm1*. (D) H3K9me detected by ChIP. The format is as shown in Fig 5. H1 and L1 regions shown above were amplified. H1 region showed robust signal for both H3K9me1 and H3K9me2, while the H3Kme2 signal is weaker in L1. The difference is consistent with results by Stroud et al (2014) that binding of CMT2 to H3K9me1 is weaker than that of CMT3 [11]. Results for other loci (H2, H3, L2, L3) are shown in S7 Fig.
(TIF)

S7 Fig H3K9me in the CHG hypermethylated loci (continued from S26). (A-D) Genome browser views of CHH and CHG methylation around *AT2G15930* (A), *AT3G08760* (B), *AT3G04765* (C), and *AT3G64850* (D) loci. Level of ectopic CHH methylation is relatively high in H2 and H3 regions but low in L2 and L3 regions. (E) H3K9me of WT and *ddm1* mutants detected by ChIP. The format is as shown in Fig 5. Amplified regions in the examined loci are indicated in (A-D). H3 and L2 show signal for 11G #1 but not for 11G #3. That is consistent with the non-CG methylation profiling in B and C; ectopic non-CG methylation of 9G *ddm1* was found in line #1 but not in line #3. L3 region behaved like *Ta3* (shown) and other TEs; they showed H3K9me signals in wild type, which is lost in *ddm1* mutants.
(TIF)

S8 Fig Hypermethylation occurred reproducibly at specific genes during independent repeated self-pollinations of *ddm1* mutants. (A) Three contexts of methylation were examined for WT, 1G and 9G *ddm1* mutant plants for genes hypermethylated in each of the contexts. Unlike Fig 3D, hypermethylated genes were selected based on difference in DNA methylation levels between WT and 9G *ddm1*. (B) Association of genes hypermethylated in each of the four lines of 9G *ddm1* plants. In each of the four lines, 1,000 genes with the largest increase of cytosine methylation were selected. CG, CHG, and CHH contexts are separately shown. “Expected” values were calculated assuming no association (random binominal distribution). Excess of “Observed” values reflects a strong association of the hypermethylated genes in four independently self-pollinated lines. Strong association was found for all three contexts of methylation.
(TIF)

S9 Fig Coordinated remethylation of TEs during self-pollinations of *ddm1*. (A) Change of DNA methylation for CHG-hypermethylated TEGs (9G *ddm1* – 1G *ddm1*) and CHH-hypermethylated TEGs (9G *ddm1* - 1G *ddm1*), compared to all TEGs shown as controls. Three contexts of sites show coordinated hypermethylation in 9G. (B) Pie charts of numbers of non-CG hypermethylated TEGs in each family of TEs shown in (A). TEGs were classified according to the family of the corresponding TE. Gypsy elements are over-represented for hypermethylation for both CHG and CHH sites.
(TIF)

S10 Fig Profiles for remethylation of TEs during self-pollinations of *ddm1*. Pattern of CHG (A) and CHH (B) methylation over TEGs are shown for each of TE families. Gypsy show strong peak outside transcription termination site for both CHG and CHH contexts.
(TIF)

S11 Fig Distribution of the effect of *ddm1* mutation among the TE families. Distribution of methylation change was shown for each of TE families for the CG (A), CHG (B) and CHH (C) contexts of methylation.
(TIF)

S12 Fig DNA methylation level in 2G *ddm1* plants. Methylation level of cytosine was compared for each transcription unit. The top half shows effects in three different 1G *ddm1* plants, while the bottom half shows effects in four different 2G *ddm1* plants. CG (A), CHG (B), and CHH (C) contexts are separately shown. Each of the 2G plants was originated from independent 1G *ddm1* plants. “WT” is a *DDM1/DDM1* plant segregating as a sibling of the 1G *ddm1/ddm1* plants.
(TIF)

S13 Fig Ectopic non-CG methylation occurring in 2G *ddm1* was slow. (A) Genome browser views of CHG methylation at *AT1G73177* (*BONSAI*) locus. Spread of CHG methylation from the LINE to *BONSAI* gene was still modest in the 2G *ddm1* compared to the 9G *ddm1*. (B) Change of CHG methylation level for genes hypermethylated in 9G *ddm1*. Results are shown for the Experiment #1 (WT, 1G *ddm1*, and 9G *ddm1*) (Fig 3A) and the Experiment #2 (WT, 1G *ddm1*, and 2G *ddm1*) (S12B Fig). The value in the right, “Theoretical prediction of 9G *ddm1*”, was calculated by extrapolating signals for 1G and 2G *ddm1* in the experiment #2. In other words, values were calculated by B + (B – A) x 7, where A and B are signals for 1G *ddm1* and 2G *ddm1* in the experiment #2. The value is much less than that in 9G *ddm1* in the experiment #1, suggesting that the ectopic hypermethylation proceed much slower in the initial generations than in later generations.
(TIF)

S14 Fig Enrichment of various histone modifications around the DMRs (9G *ddm1* - 1G *ddm1)*. Normalized scores were calculated using the 100 thousand regions chosen randomly from the genome. Only DMRs that overlapped with genes were used; Each DMR was aligned according to the orientation of the corresponding gene. ChIP-seq data was obtained from GEO (GSE28398 [72]).
(TIF)

S15 Fig Progressive accumulation of non-CG methylation in *ibm1* mutants. Patterns of DNA methylation over total genes and TEGs are shown for WT, 1G *ibm1*, and 3G *ibm1*. 1G *ibm1* plants are progeny of an *IBM1*/*ibm1* heterozygote. Their *ibm1*/*ibm1* siblings were self-pollinated twice and the progenies were used as 3G *ibm1*.
(TIF)

S16 Fig Comparison of CHG methylation level in DMRs. (A) Venn diagram for data shown in Fig 6B. (B) Heatmap of CHG methylation for the DMRs shown in A.
(TIF)

S17 Fig Change of DNA methylation in epiRILs. For seven loci, changes of local DNA methylation level were plotted against the global hypomethylation as shown in Fig 7. Dots of light green and red are values for parental *DDM1* and 4G *ddm1* plants, respectively. Strong positive correlation was found in six out of seven loci examined (panels A, B, C, E, F, and G, but not in D; S1 Table).
(TIF)

S18 Fig The haplotypes of epiRILs that showed increase of cytosine methylation (*AT1G73177*). In this figure and S19–S23 Figs, inference of the haplotypes in epiRILs are shown for all five chromosomes for each of the loci shown in S17 Fig. WT/*ddm1* haplotype was determined by stably hypomethylated markers. Three loci (*AT1G73177*, *AT2G39540* and *AT1G03660*) are localized near telomere with only one reference marker flanking them. In the other loci, every plants showed consistent haplotype for the markers flanking both sides, except for line 5 (epiRIL98) of *AT4G30975*, with the two flanking markers showing different haplotypes. We could not find a locus consistently derived from *ddm1* parent in all of the plants showing the high level of ectopic hypermethylation in the six loci
(TIF)

S19 Fig The haplotypes of epiRILs that showed increase of cytosine methylation (*AT5G52480*). See legend of S18 Fig for details.
(TIF)

S20 Fig The haplotypes of epiRILs that showed increase of cytosine methylation (*AT5G35510*). See legend of S18 Fig for details.
(TIF)

S21 Fig The haplotypes of epiRILs that showed increase of cytosine methylation (*AT4G30975*). See legend of S18 Fig for details.
(TIF)

S22 Fig The haplotypes of epiRILs that showed increase of cytosine methylation (*AT2G39540*). See legend of S18 Fig for details.
(TIF)

S23 Fig The haplotypes of epiRILs that showed increase of cytosine methylation (*AT1G03660*). See legend of S18 Fig for details.
(TIF)

S24 Fig Ectopic non-CG methylation found in epiRIL98. Genome browser views of CHG methylation in the *BONSAI* (*AT1G73177*) locus (A), and in *AT5G16880* locus (B). The latter locus has a high level of CG methylation (C). For both loci, CHG methylation increased in the 9G *ddm1* plants and also in epiRIL98.
(TIF)

S25 Fig Ectopic non-CG methylation found in WT-like chromosome in epiRIL98. Genome browser views of CHG methylation in *AT3G22980* locus (A) and *AT1G35220* locus (B). These loci are in the WT-like haplotype in epiRIL98.
(TIF)

S26 Fig The *ddm1*–induced ectopic methylation is not due to repression of DNA demethylase gene *ROS1*. (A) *ROS1* gene expression of 4G *ddm1*, and epiRIL98 compared to WT. Closed circles and error bars indicate the mean and SD of the signals of the probes for *ROS1* locus. Although the expression is reduced in the 4G *ddm1*, it was almost normal for epiRIL98. The data were obtained from GEO (GSE37106 [46]). (B) Overlap between the genes hyper-methylated in *ros1-dml2-dml3* triple mutant (data from Penterman et al., 2007 [76]) and the genes hyper-methylated in CHG context during self-pollination of *ddm1*.
(TIF)

S27 Fig The difference between the effect of self-pollination of *ddm1* mutation and that of *met1* mutation. Overlap of regions CHG hyper-methylated in *met1*, 3G *ibm1* and 9G *ddm1*. DMRs between 9G and 1G *ddm1* (blue), between 1G *met1* and wild type (green; Data were obtained from GEO (GSE39901 [24]), and between 3G *ibm1* and wild type (red) are shown.
(TIF)
