# Supplementary figures and images for: Genome-Wide Negative Feedback Drives Transgenerational DNA Methylation Dynamics in Arabidopsis
Source: PLoS Genet. 2015 Apr 22;11(4):e1005154. doi: 10.1371/journal.pgen.1005154 (PMC4406451; doi:10.1371/journal.pgen.1005154)

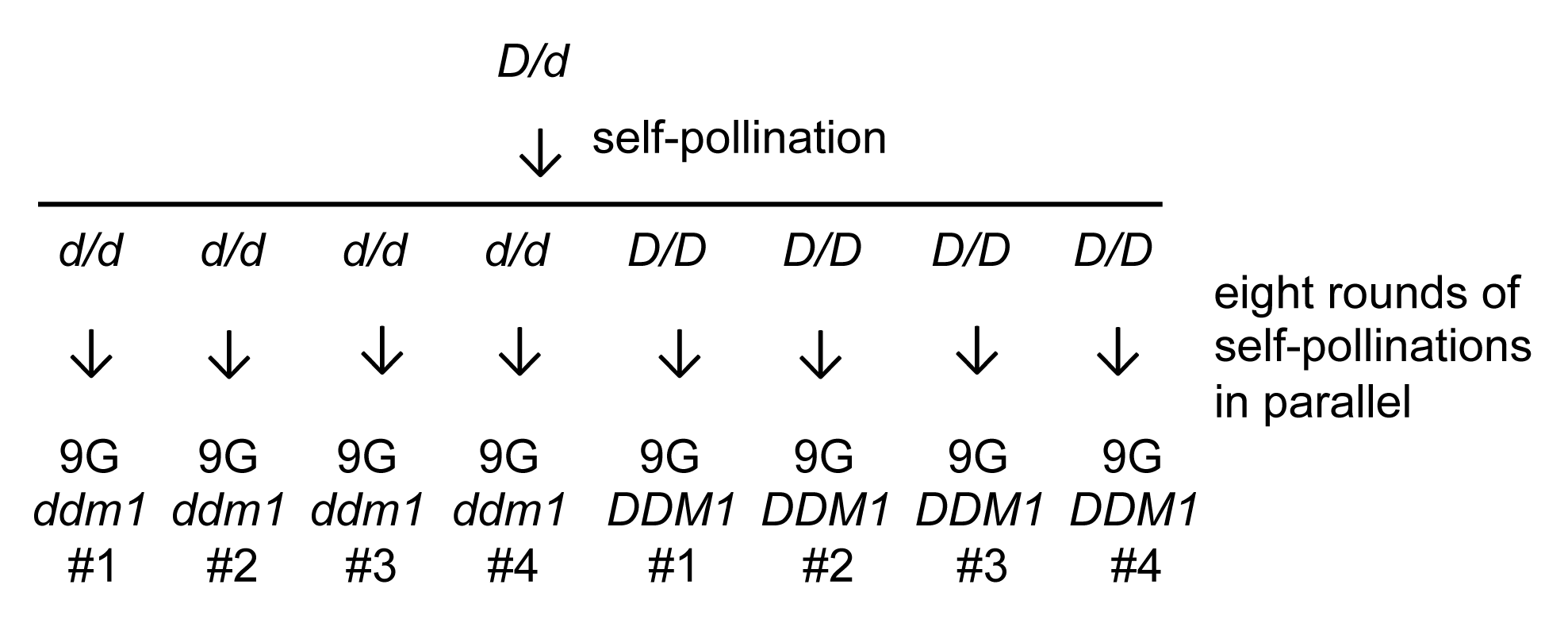

Supplement: S1 Fig — Genetic scheme of the production of self-pollinated ddm1 and control DDM1 lines. The parental DDM1/ddm1 (shown as D/d) is generated by backcrossing original ddm1 mutant to wild type six times in the heterozygous state. In the self-pollinated progeny of the heterozygote, multiple ddm1/ddm1 (d/d) and DDM1/DDM1 (D/D) plants were selected and self-pollinated eight times independently to generate 9G ddm1 and control 9G DDM1 plants (TIF) [file pgen.1005154.s003.tif]

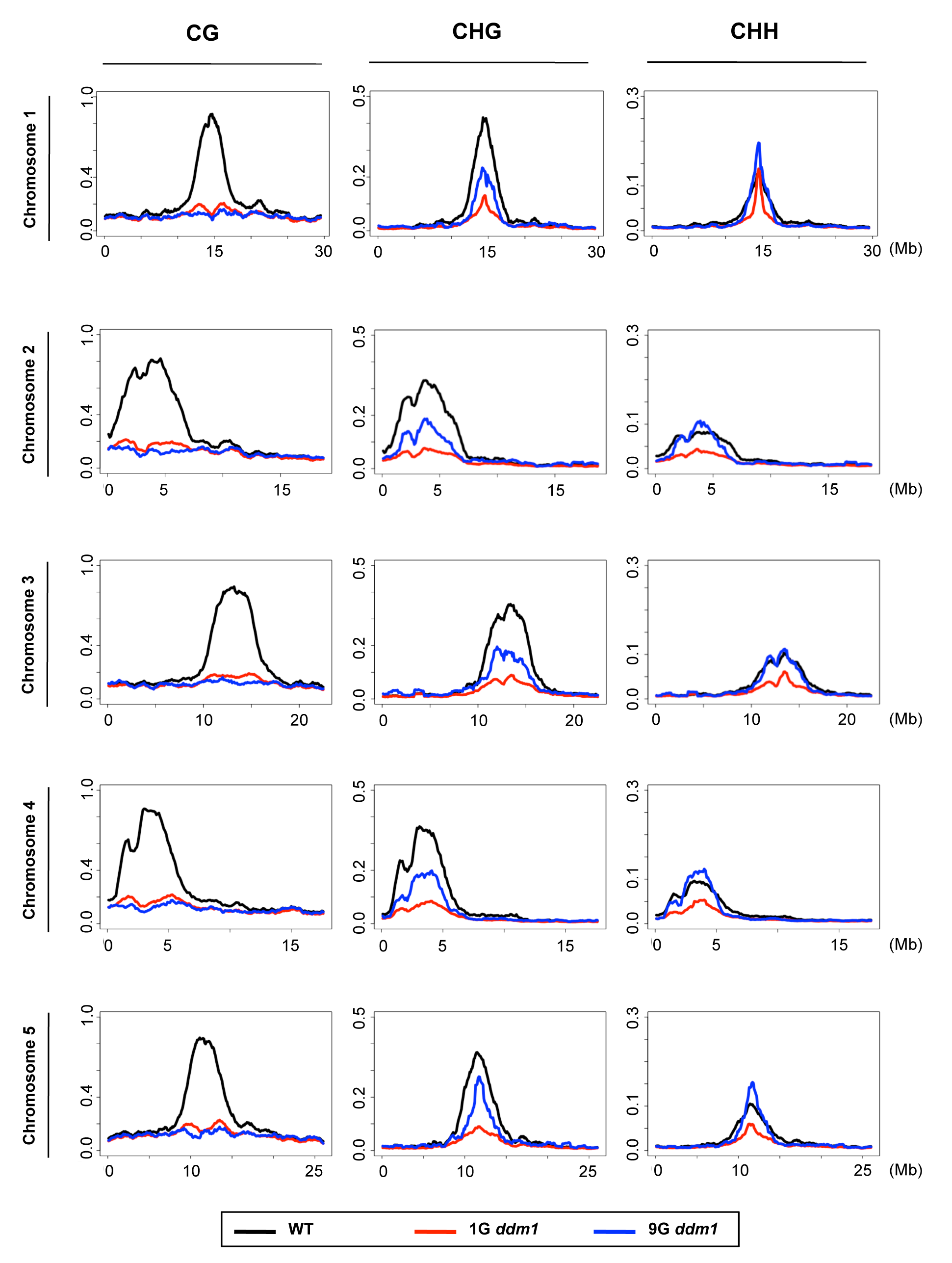

Supplement: S2 Fig — Cytosine methylation levels are shown for the three contexts, CG, CHG and CHH, with the sliding windows of 1Mb. (TIF) [file pgen.1005154.s004.tif]

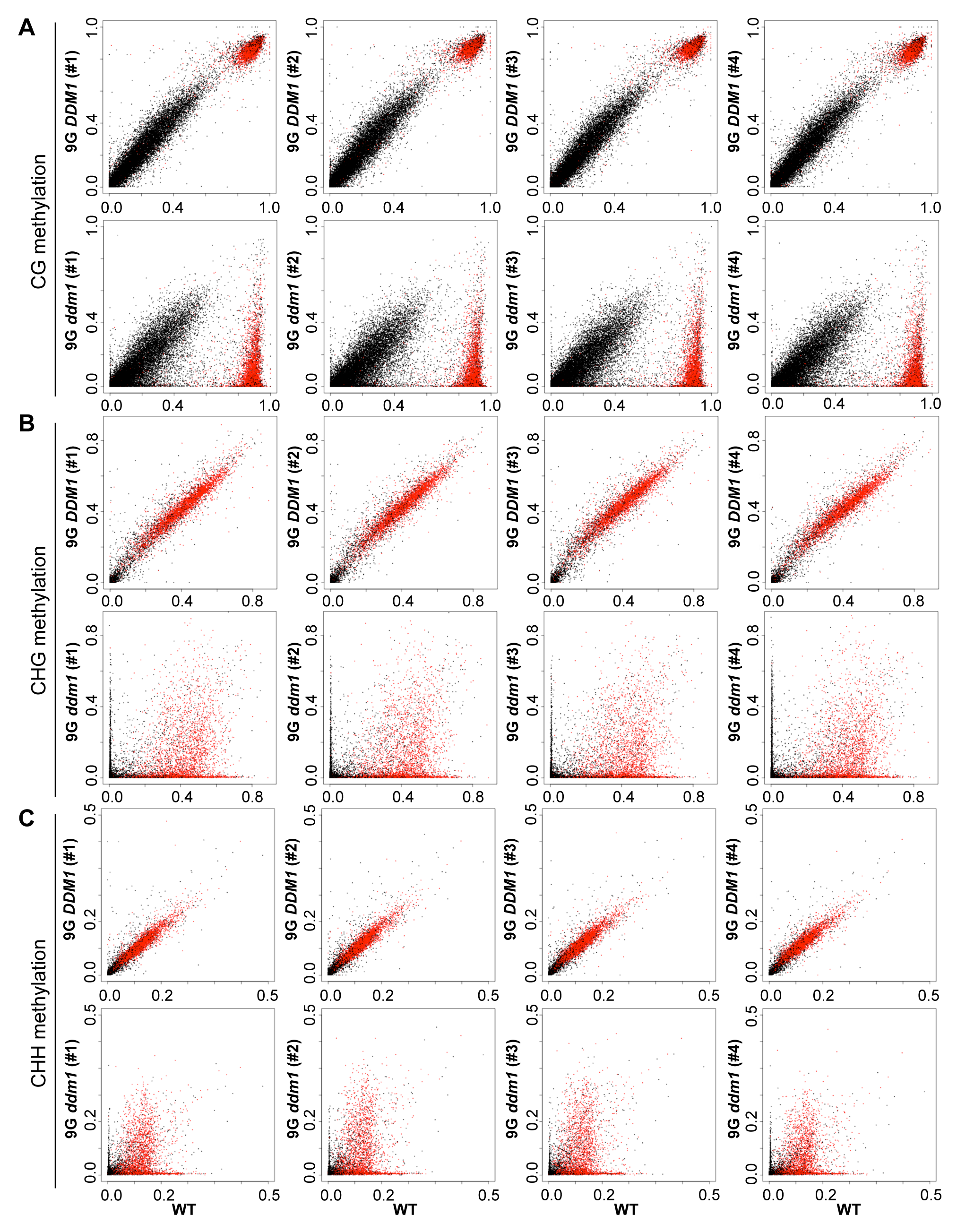

Supplement: S3 Fig — Methylation level of cytosine was compared for each transcription unit between 9G DDM1/DDM1 plants and WT in the CG (A), CHG (B), and CHH (C) contexts. The format is as shown in Fig 2A. Each of the 9G plants was originated from independent self-pollinations (S1 Fig). “WT” is a DDM1/DDM1 plant segregating as a sibling of the 1G ddm1/ddm1 plants. (TIF) [file pgen.1005154.s005.tif]

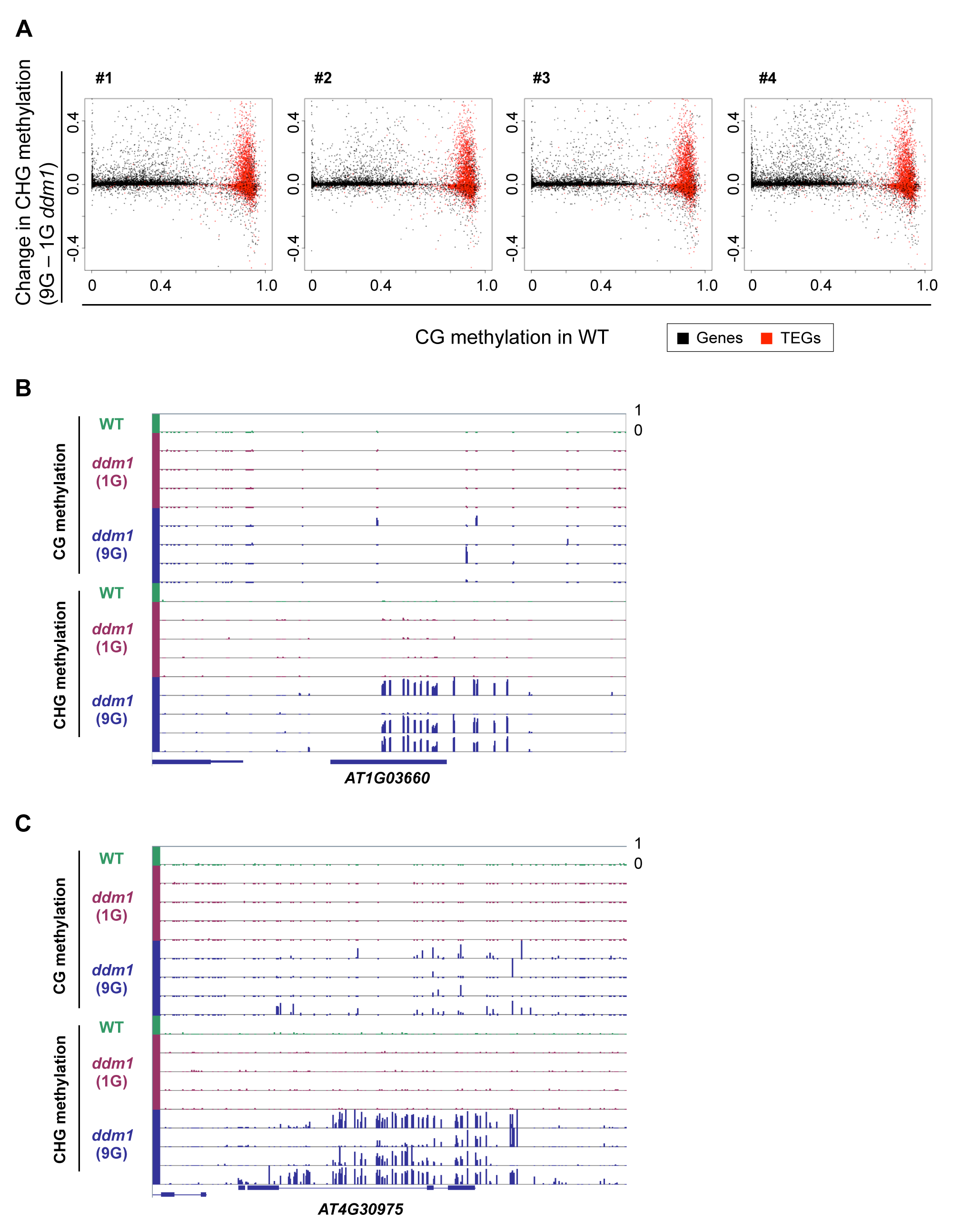

Supplement: S4 Fig — Methylation level of cytosine was compared for each transcription unit. (A) Change in CHG methylation between 1G and 9G ddm1 plotted against CG methylation level in WT. Although many of the body-methylated genes show the ectopic CHG methylation, substantial number of unmethylated genes also showed the ectopic CHG methylation. (B-C) Two examples of genes without CG methylation in WT, but gained CHG methylation in 9G ddm1. In these loci, the 9G ddm1 also showed low level of ectopic CG methylation. The ectopic CG hypermethylation accompanied by non-CG methylation is also found in other loci [39]. (TIF) [file pgen.1005154.s006.tif]

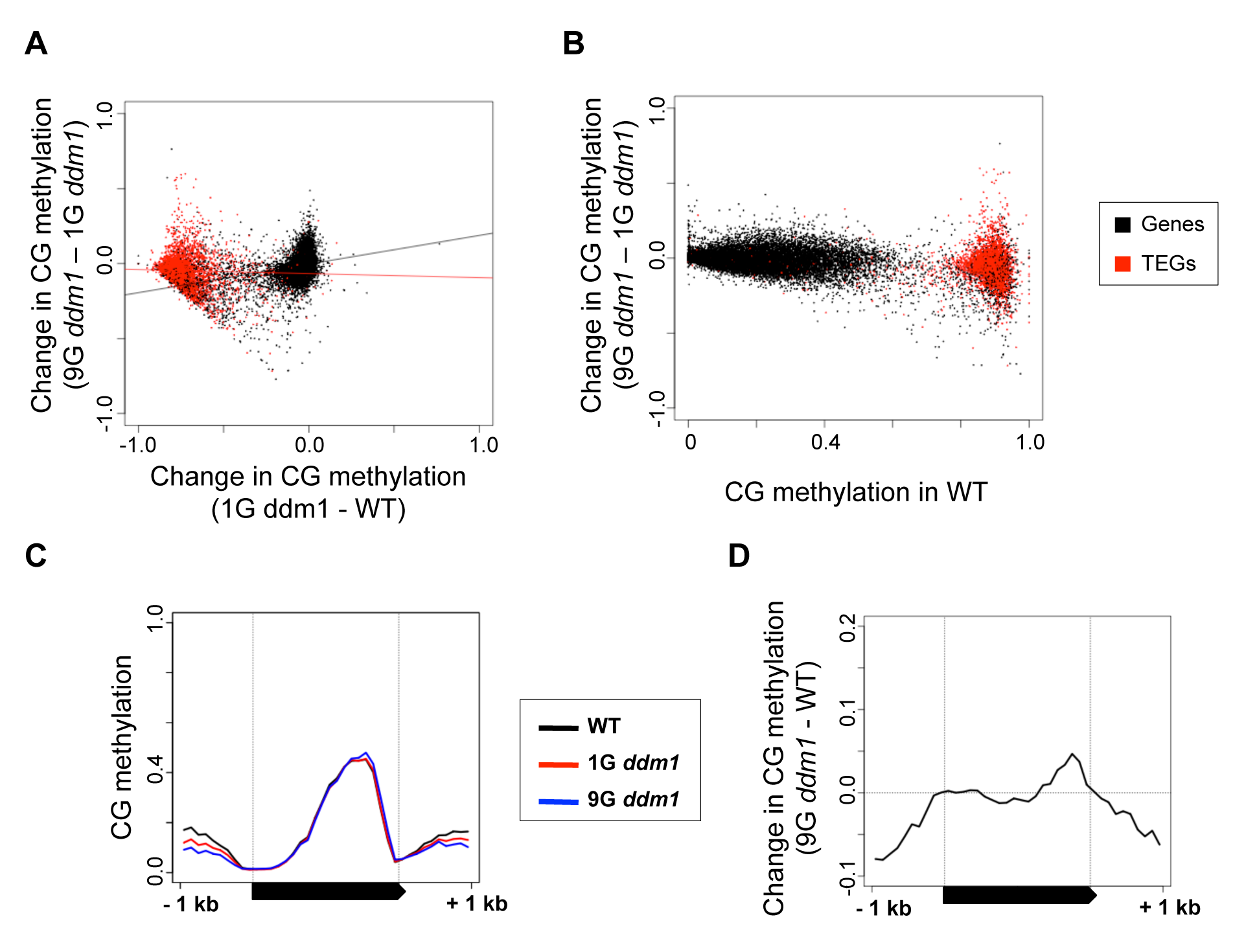

Supplement: S5 Fig — Methylation level of cytosine was compared for each transcription unit. (A) Comparison of two changes of CG methylation, (from WT to 1G ddm1) and (from 1G ddm1 to 9G ddm1). Regression lines for the data of genes (black) and TEGs (red) were calculated using least square method. The two changes correlate negatively in TEs reflecting that some TEs loose methylation in 1G ddm1 but regained that in 9G ddm1. In contrast, The correlation was positive in genes, suggesting that many genes accumulate CG methylation in 9G ddm1, even though they do not loose methylation in 1G ddm1. (B) Change in CG methylation from 1G to 9G ddm1 plotted against CG methylation level in WT. Some genes accumulate CG methylation in 9G, even if they do not have CG methylation in WT. The results are analogous to that in S4A Fig; ectopic CG and non-CG methylation can accumulate in 9G ddm1 even for genes without CG methylation in wild type. (C) Patterns of CG methylation for the genes CG hypermethylated in 9G ddm1 (genes defined as “hypermethylated” for at least three lines in S8B Fig) compared among WT, 1G ddm1 and 9G ddm1. (D) The pattern of difference of CG methylation between WT and 9G ddm1 over the genes hypermethylated in CG context. The peak of the increase was shifted to 3’ region, compared to peak in CG body methylation shown in (C). (TIF) [file pgen.1005154.s007.tif]

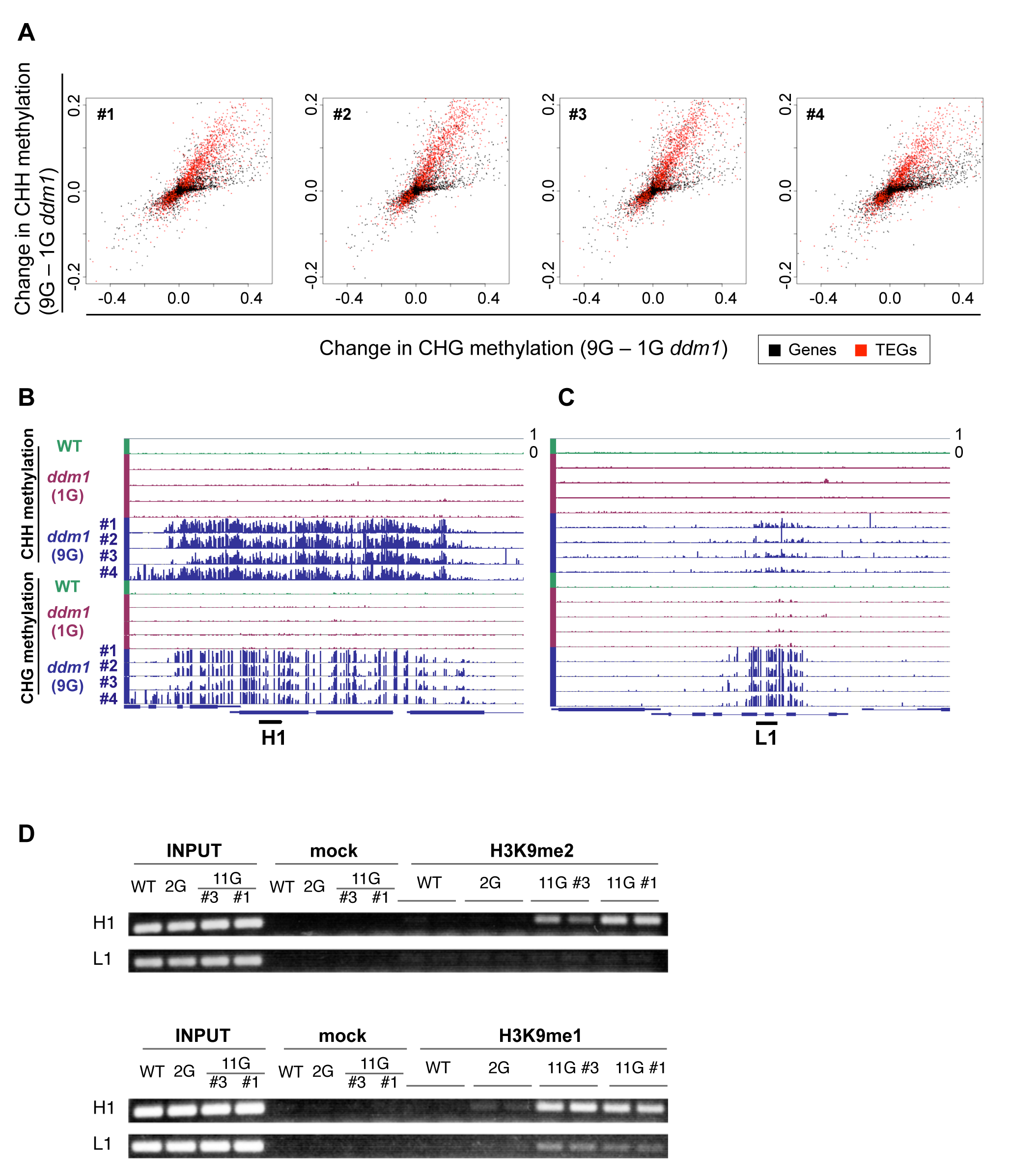

Supplement: S6 Fig — (A) Change in CHG methylation and CHH methylation during self-pollination of ddm1. TEGs (red) tend to show more CHH methylation than genes (black) with similar level of CHG methylation. In addition, some genes show more CHH methylation than others with similar level of CHG methylation. (B-C) Genome browser views of CHH and CHG methylation around AT5G15890 (B) and AT1G06460 (C) loci. H1 and L1 are regions amplified in (D). H1 and L1 regions were selected for relatively high and low level of ectopic CHH methylation, respectively (details in Materials and Methods section). Both were CHG hypermethylated in 9G ddm1. (D) H3K9me detected by ChIP. The format is as shown in Fig 5. H1 and L1 regions shown above were amplified. H1 region showed robust signal for both H3K9me1 and H3K9me2, while the H3Kme2 signal is weaker in L1. The difference is consistent with results by Stroud et al (2014) that binding of CMT2 to H3K9me1 is weaker than that of CMT3 [11]. Results for other loci (H2, H3, L2, L3) are shown in S7 Fig. (TIF) [file pgen.1005154.s008.tif]

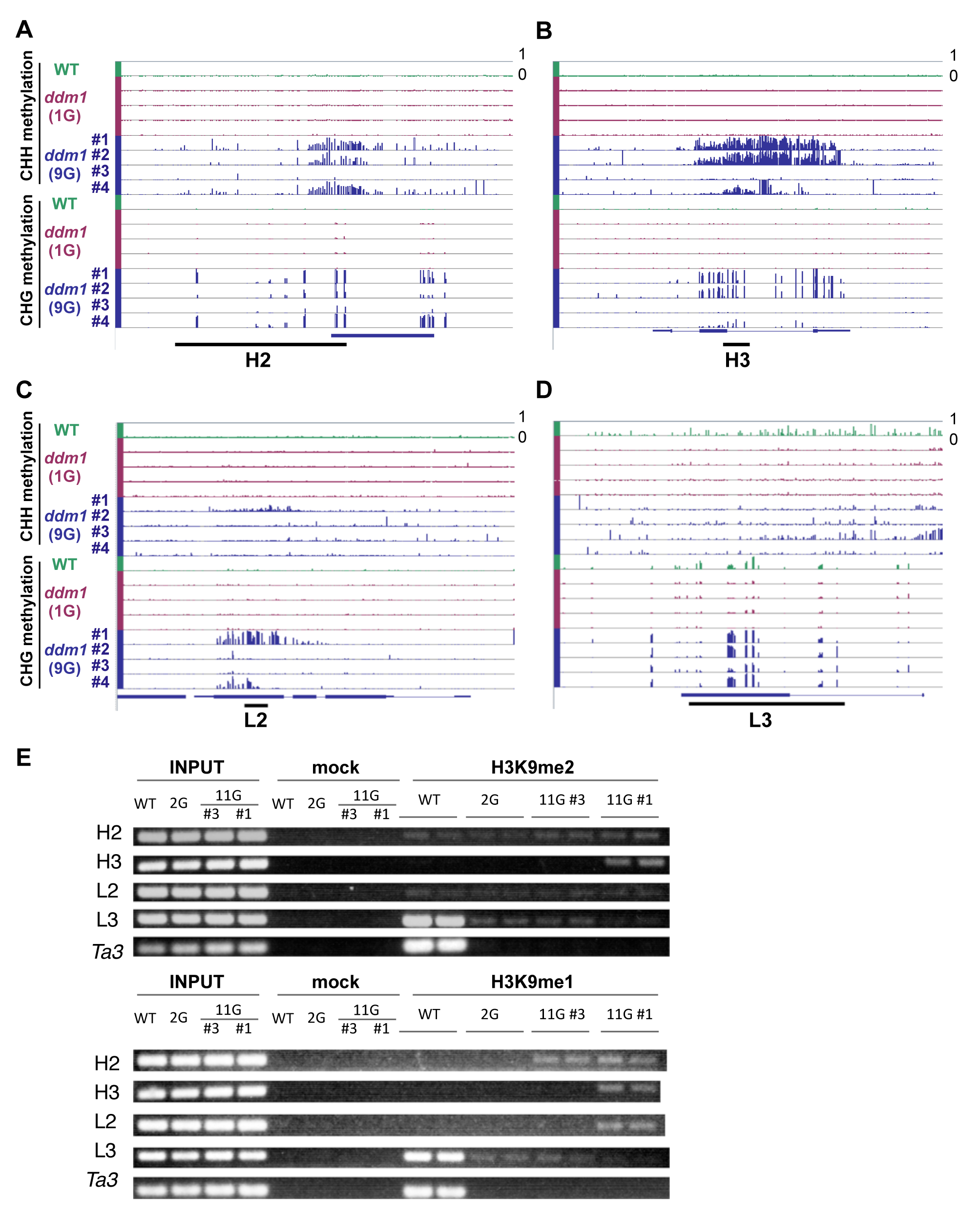

Supplement: S7 Fig — (A-D) Genome browser views of CHH and CHG methylation around AT2G15930 (A), AT3G08760 (B), AT3G04765 (C), and AT3G64850 (D) loci. Level of ectopic CHH methylation is relatively high in H2 and H3 regions but low in L2 and L3 regions. (E) H3K9me of WT and ddm1 mutants detected by ChIP. The format is as shown in Fig 5. Amplified regions in the examined loci are indicated in (A-D). H3 and L2 show signal for 11G #1 but not for 11G #3. That is consistent with the non-CG methylation profiling in B and C; ectopic non-CG methylation of 9G ddm1 was found in line #1 but not in line #3. L3 region behaved like Ta3 (shown) and other TEs; they showed H3K9me signals in wild type, which is lost in ddm1 mutants. (TIF) [file pgen.1005154.s009.tif]

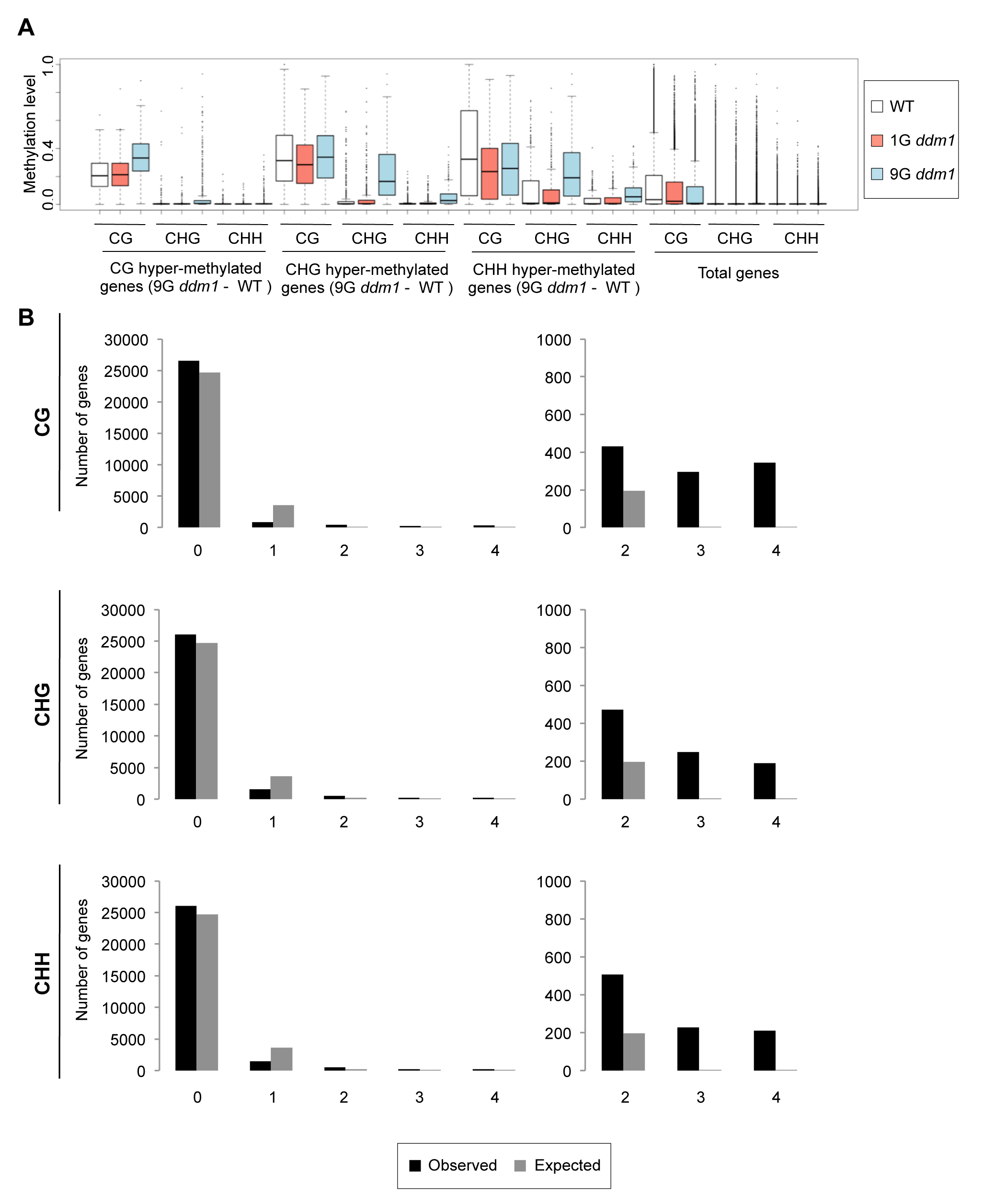

Supplement: S8 Fig — (A) Three contexts of methylation were examined for WT, 1G and 9G ddm1 mutant plants for genes hypermethylated in each of the contexts. Unlike Fig 3D, hypermethylated genes were selected based on difference in DNA methylation levels between WT and 9G ddm1. (B) Association of genes hypermethylated in each of the four lines of 9G ddm1 plants. In each of the four lines, 1,000 genes with the largest increase of cytosine methylation were selected. CG, CHG, and CHH contexts are separately shown. “Expected” values were calculated assuming no association (random binominal distribution). Excess of “Observed” values reflects a strong association of the hypermethylated genes in four independently self-pollinated lines. Strong association was found for all three contexts of methylation. (TIF) [file pgen.1005154.s010.tif]

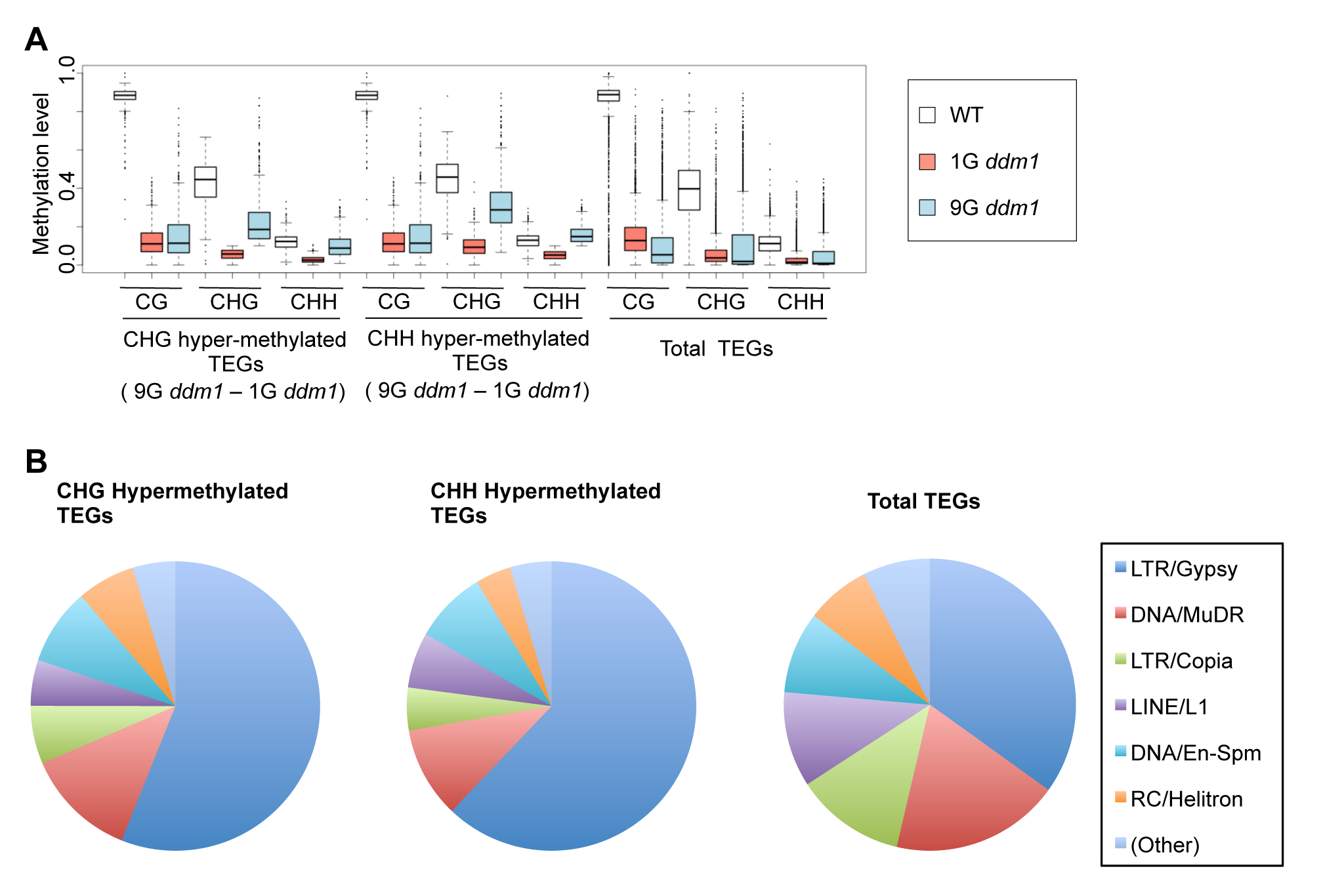

Supplement: S9 Fig — (A) Change of DNA methylation for CHG-hypermethylated TEGs (9G ddm1 – 1G ddm1) and CHH-hypermethylated TEGs (9G ddm1 - 1G ddm1), compared to all TEGs shown as controls. Three contexts of sites show coordinated hypermethylation in 9G. (B) Pie charts of numbers of non-CG hypermethylated TEGs in each family of TEs shown in (A). TEGs were classified according to the family of the corresponding TE. Gypsy elements are over-represented for hypermethylation for both CHG and CHH sites. (TIF) [file pgen.1005154.s011.tif]

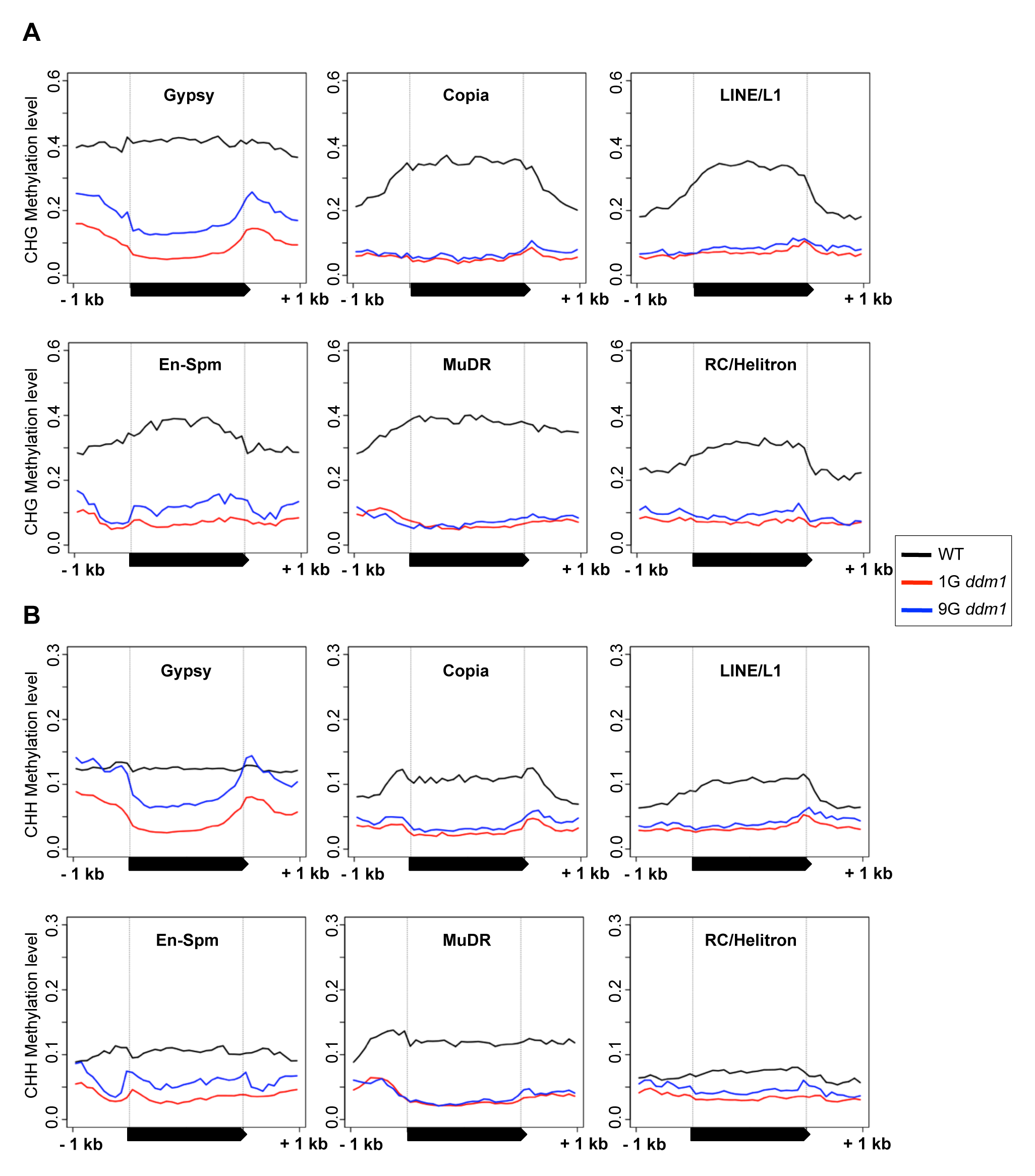

Supplement: S10 Fig — Pattern of CHG (A) and CHH (B) methylation over TEGs are shown for each of TE families. Gypsy show strong peak outside transcription termination site for both CHG and CHH contexts. (TIF) [file pgen.1005154.s012.tif]

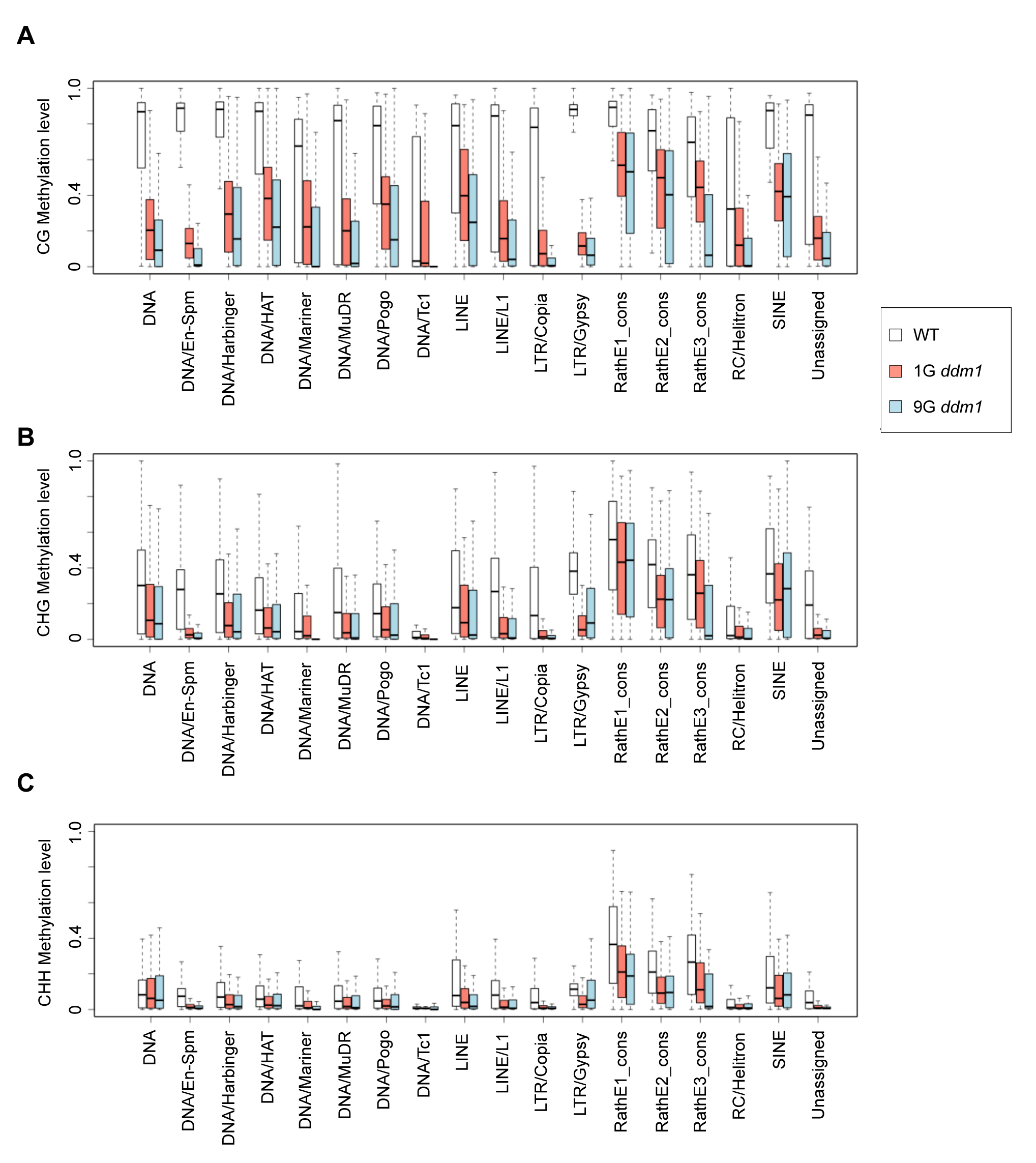

Supplement: S11 Fig — Distribution of methylation change was shown for each of TE families for the CG (A), CHG (B) and CHH (C) contexts of methylation. (TIF) [file pgen.1005154.s013.tif]

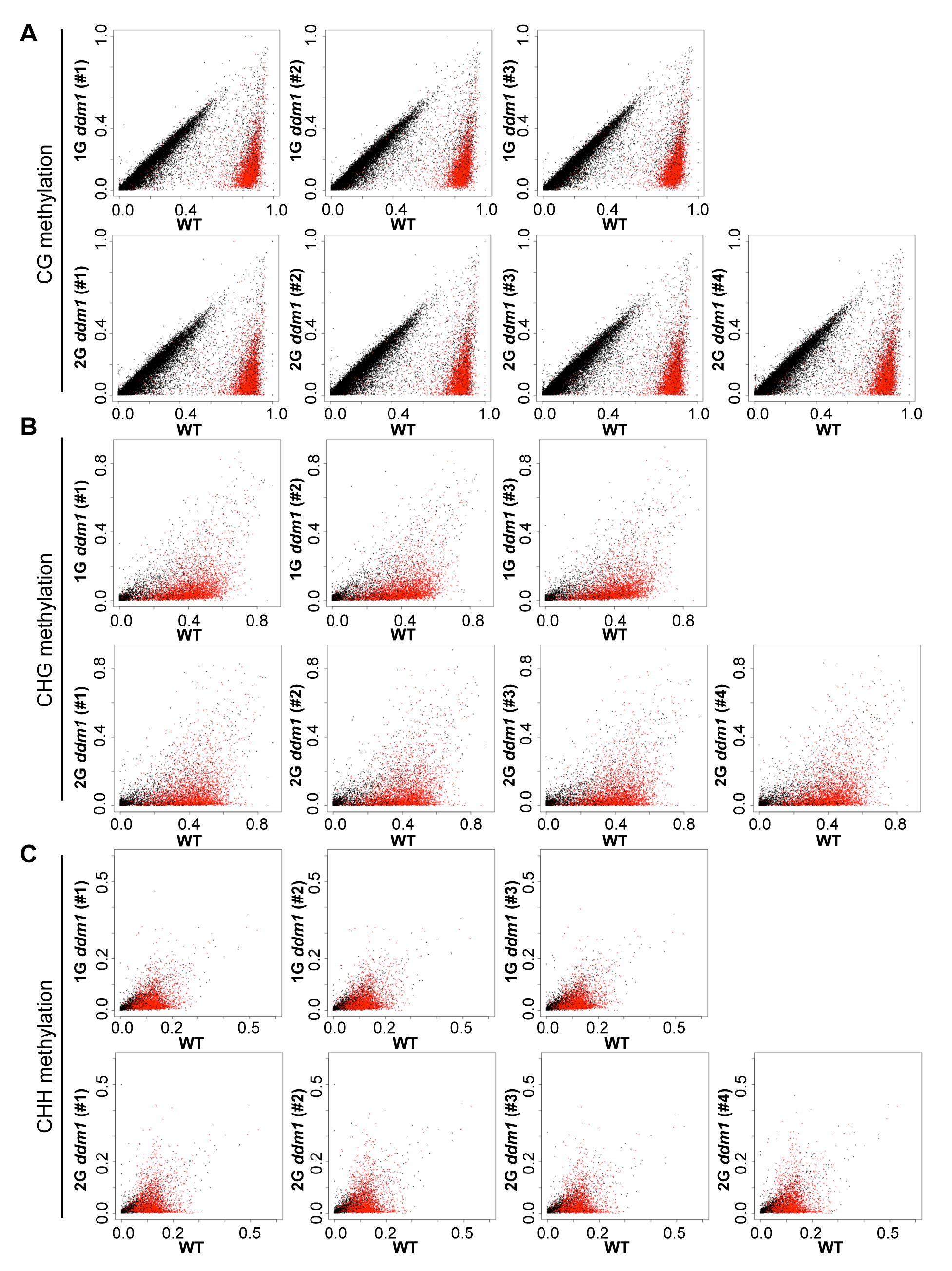

Supplement: S12 Fig — Methylation level of cytosine was compared for each transcription unit. The top half shows effects in three different 1G ddm1 plants, while the bottom half shows effects in four different 2G ddm1 plants. CG (A), CHG (B), and CHH (C) contexts are separately shown. Each of the 2G plants was originated from independent 1G ddm1 plants. “WT” is a DDM1/DDM1 plant segregating as a sibling of the 1G ddm1/ddm1 plants. (TIF) [file pgen.1005154.s014.tif]

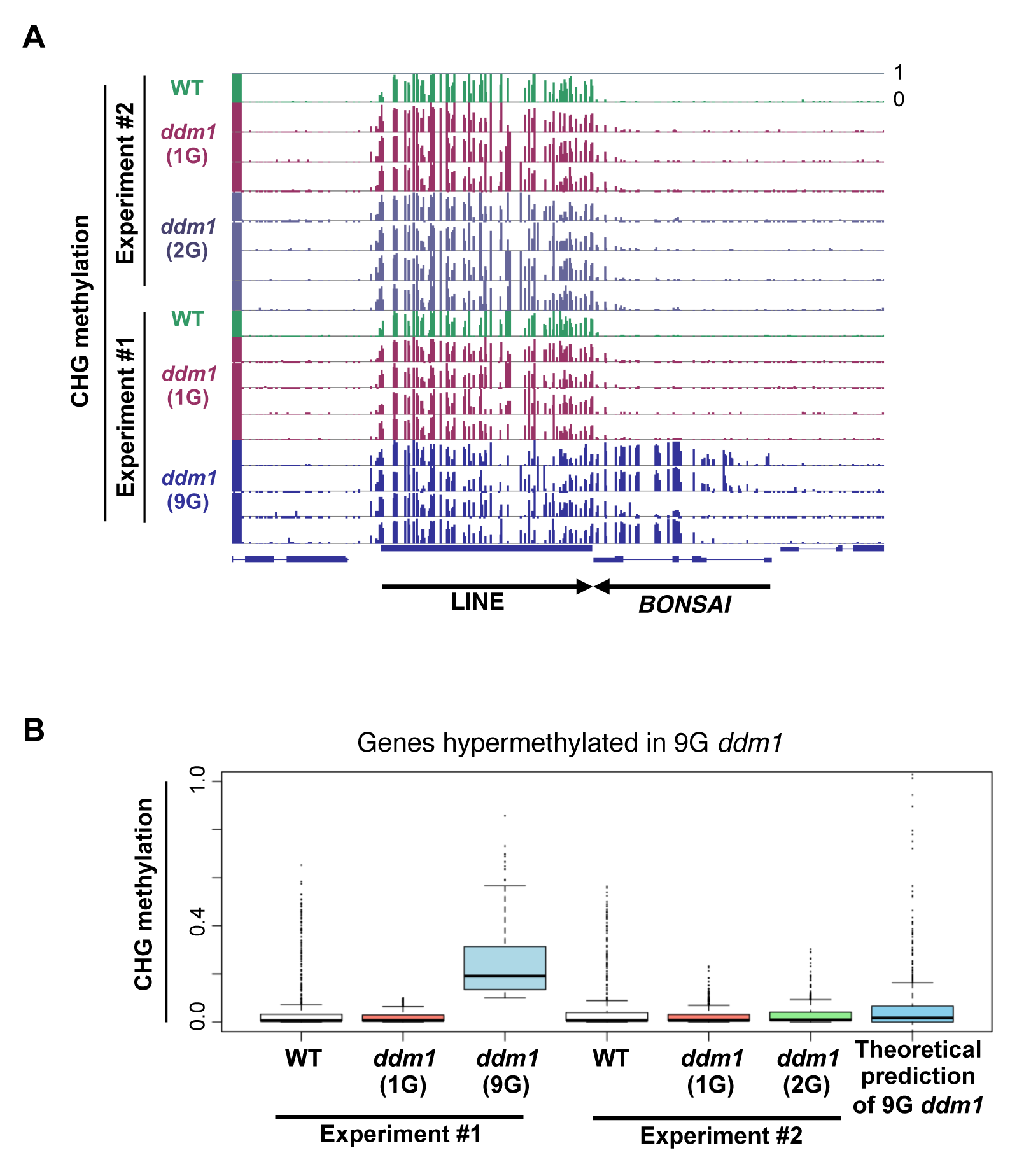

Supplement: S13 Fig — (A) Genome browser views of CHG methylation at AT1G73177 (BONSAI) locus. Spread of CHG methylation from the LINE to BONSAI gene was still modest in the 2G ddm1 compared to the 9G ddm1. (B) Change of CHG methylation level for genes hypermethylated in 9G ddm1. Results are shown for the Experiment #1 (WT, 1G ddm1, and 9G ddm1) (Fig 3A) and the Experiment #2 (WT, 1G ddm1, and 2G ddm1) (S12B Fig). The value in the right, “Theoretical prediction of 9G ddm1”, was calculated by extrapolating signals for 1G and 2G ddm1 in the experiment #2. In other words, values were calculated by B + (B – A) x 7, where A and B are signals for 1G ddm1 and 2G ddm1 in the experiment #2. The value is much less than that in 9G ddm1 in the experiment #1, suggesting that the ectopic hypermethylation proceed much slower in the initial generations than in later generations. (TIF) [file pgen.1005154.s015.tif]

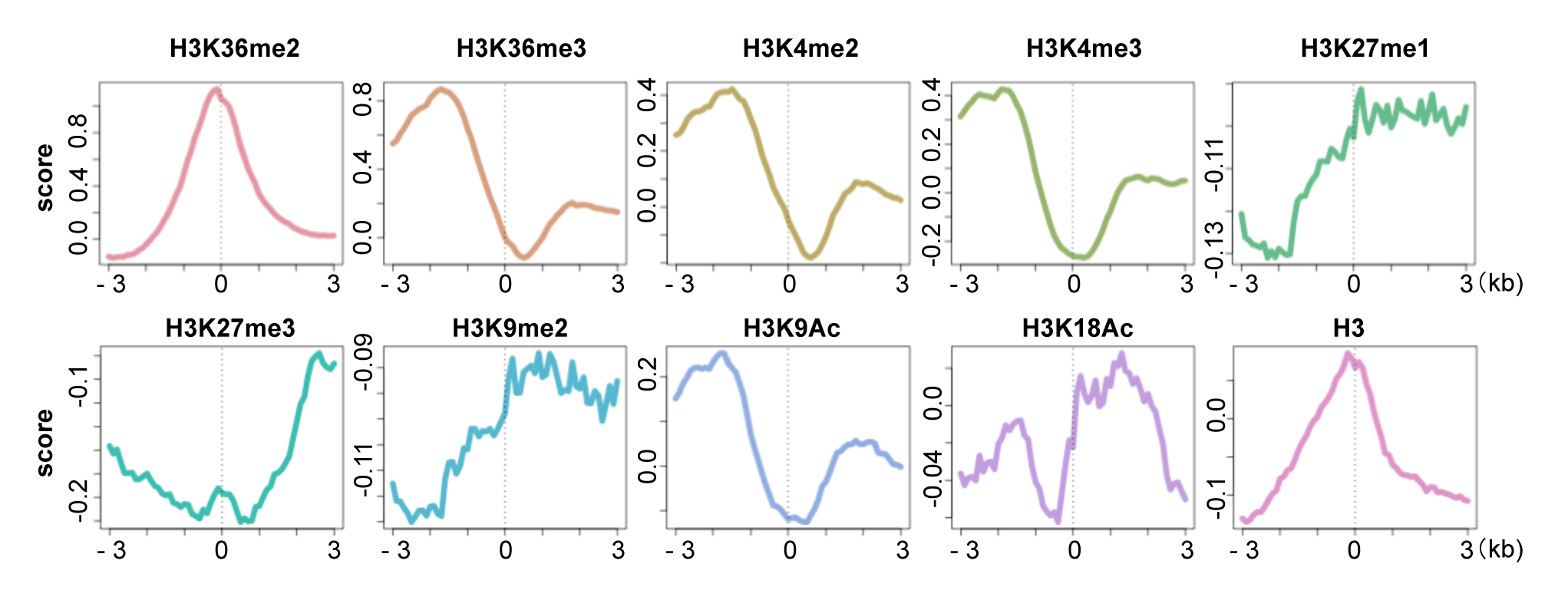

Supplement: S14 Fig — Normalized scores were calculated using the 100 thousand regions chosen randomly from the genome. Only DMRs that overlapped with genes were used; Each DMR was aligned according to the orientation of the corresponding gene. ChIP-seq data was obtained from GEO (GSE28398 [72]). (TIF) [file pgen.1005154.s016.tif]

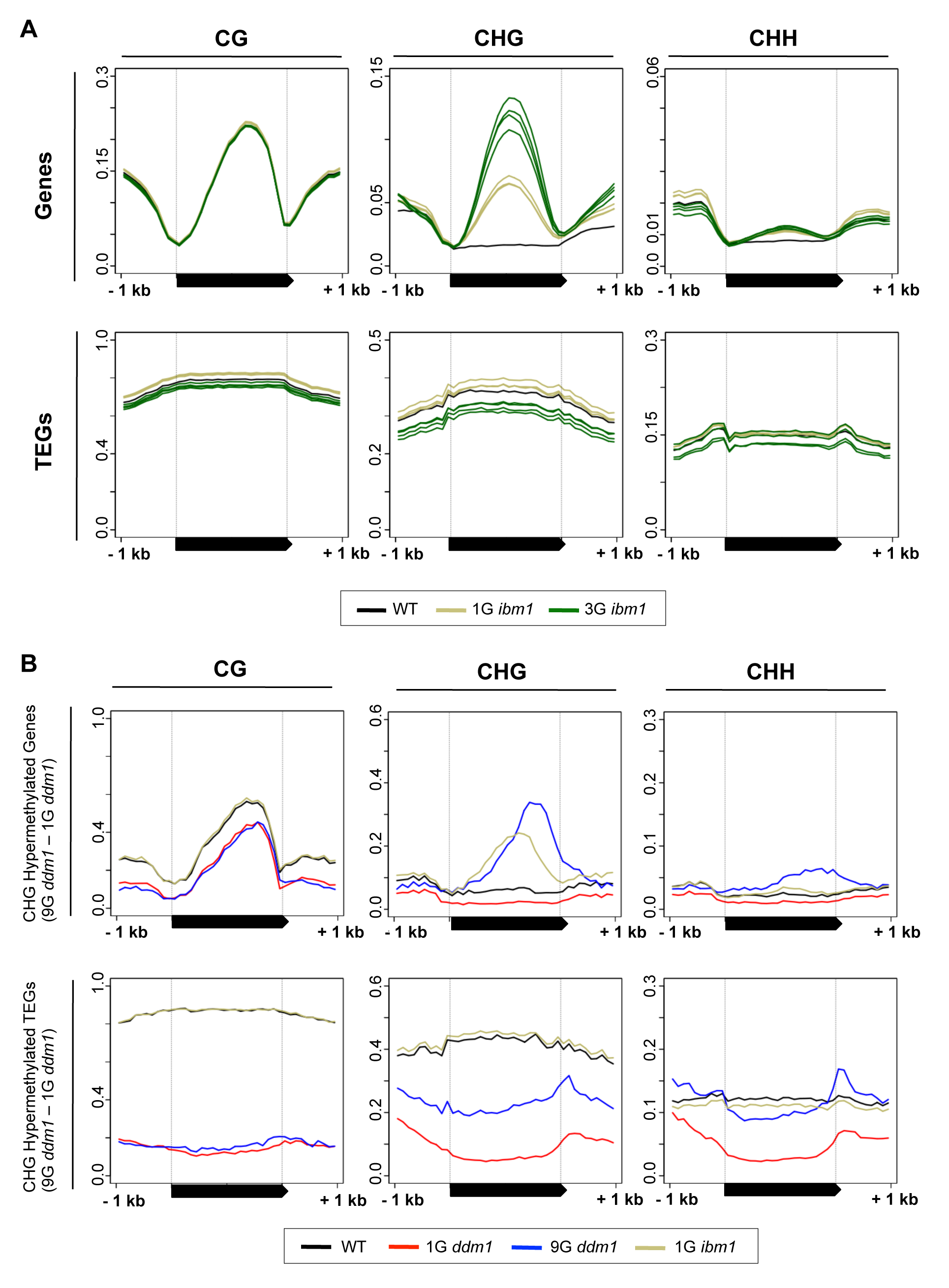

Supplement: S15 Fig — Patterns of DNA methylation over total genes and TEGs are shown for WT, 1G ibm1, and 3G ibm1. 1G ibm1 plants are progeny of an IBM1/ibm1 heterozygote. Their ibm1/ibm1 siblings were self-pollinated twice and the progenies were used as 3G ibm1. (TIF) [file pgen.1005154.s017.tif]

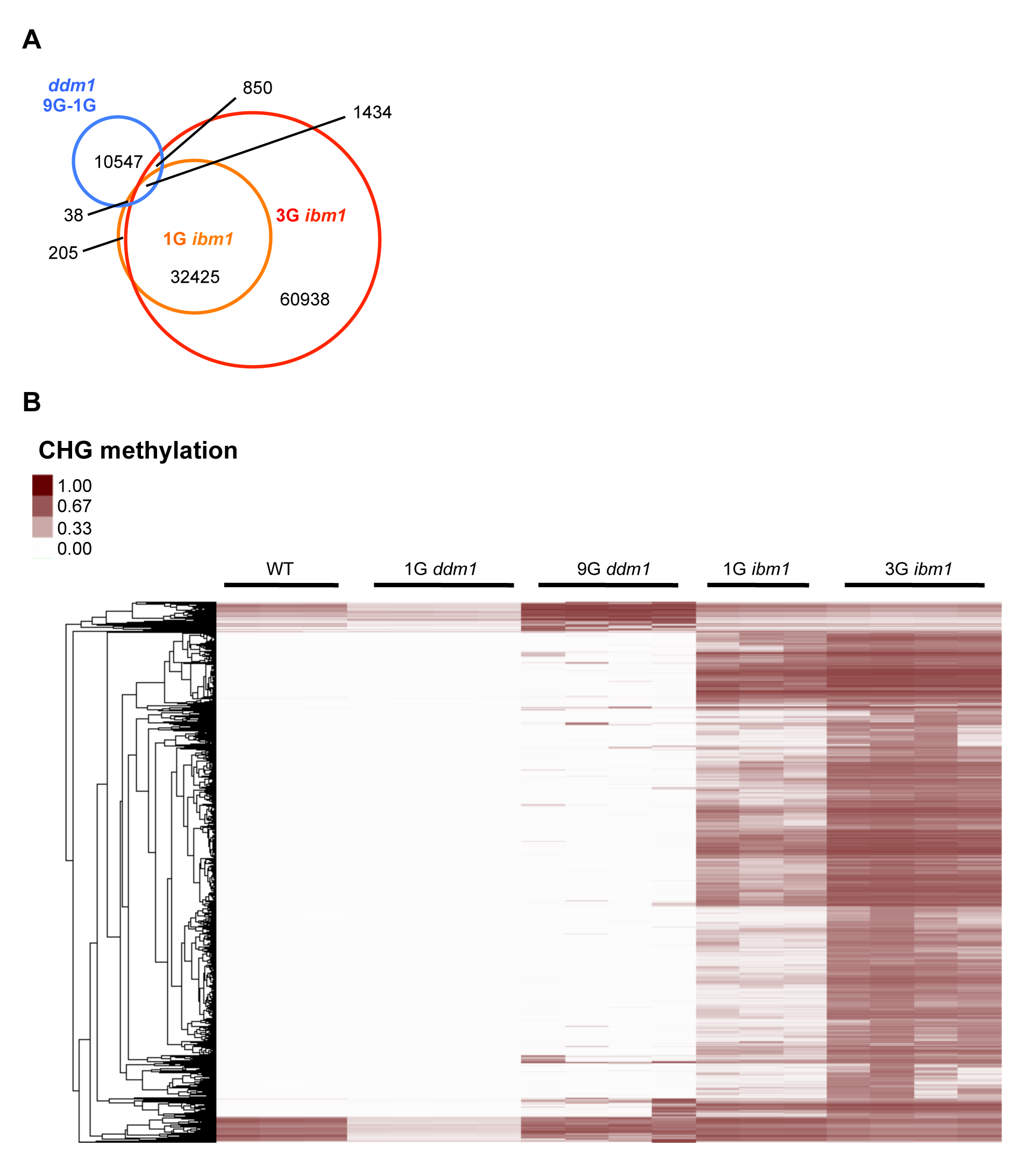

Supplement: S16 Fig — (A) Venn diagram for data shown in Fig 6B. (B) Heatmap of CHG methylation for the DMRs shown in A. (TIF) [file pgen.1005154.s018.tif]

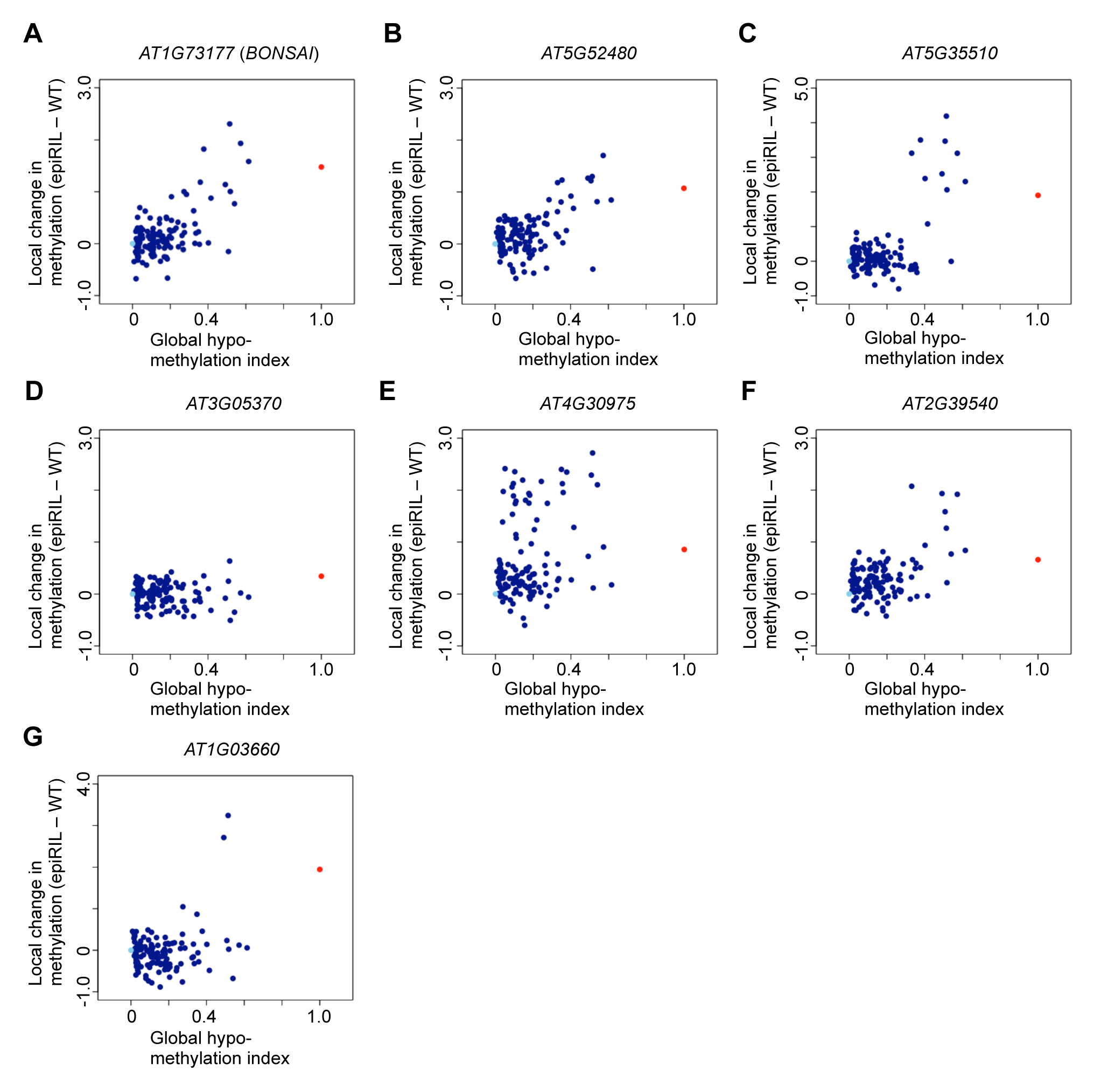

Supplement: S17 Fig — For seven loci, changes of local DNA methylation level were plotted against the global hypomethylation as shown in Fig 7. Dots of light green and red are values for parental DDM1 and 4G ddm1 plants, respectively. Strong positive correlation was found in six out of seven loci examined (panels A, B, C, E, F, and G, but not in D; S1 Table). (TIF) [file pgen.1005154.s019.tif]

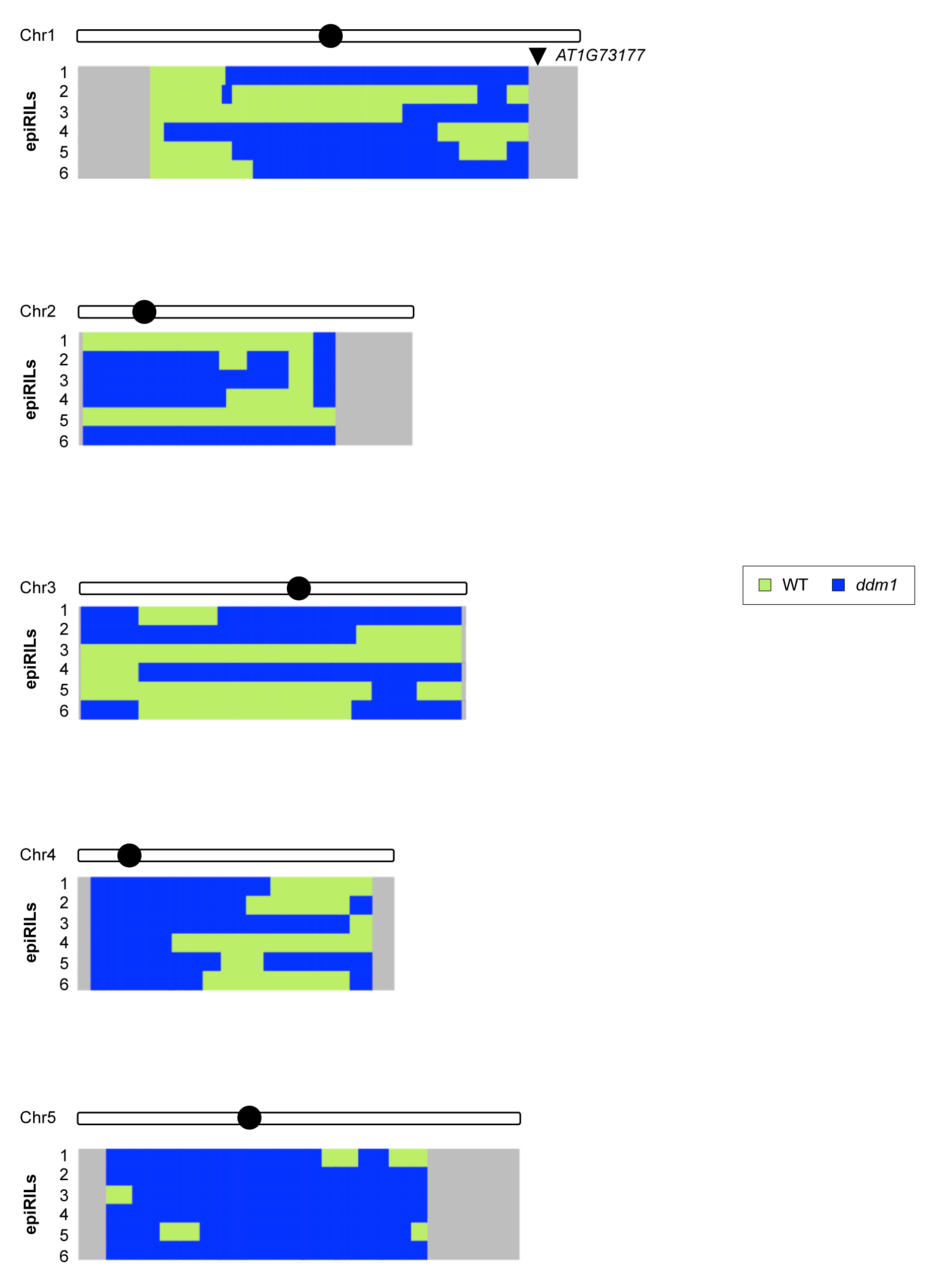

Supplement: S18 Fig — In this figure and S19–S23 Figs, inference of the haplotypes in epiRILs are shown for all five chromosomes for each of the loci shown in S17 Fig. WT/ddm1 haplotype was determined by stably hypomethylated markers. Three loci (AT1G73177, AT2G39540 and AT1G03660) are localized near telomere with only one reference marker flanking them. In the other loci, every plants showed consistent haplotype for the markers flanking both sides, except for line 5 (epiRIL98) of AT4G30975, with the two flanking markers showing different haplotypes. We could not find a locus consistently derived from ddm1 parent in all of the plants showing the high level of ectopic hypermethylation in the six loci (TIF) [file pgen.1005154.s020.tif]

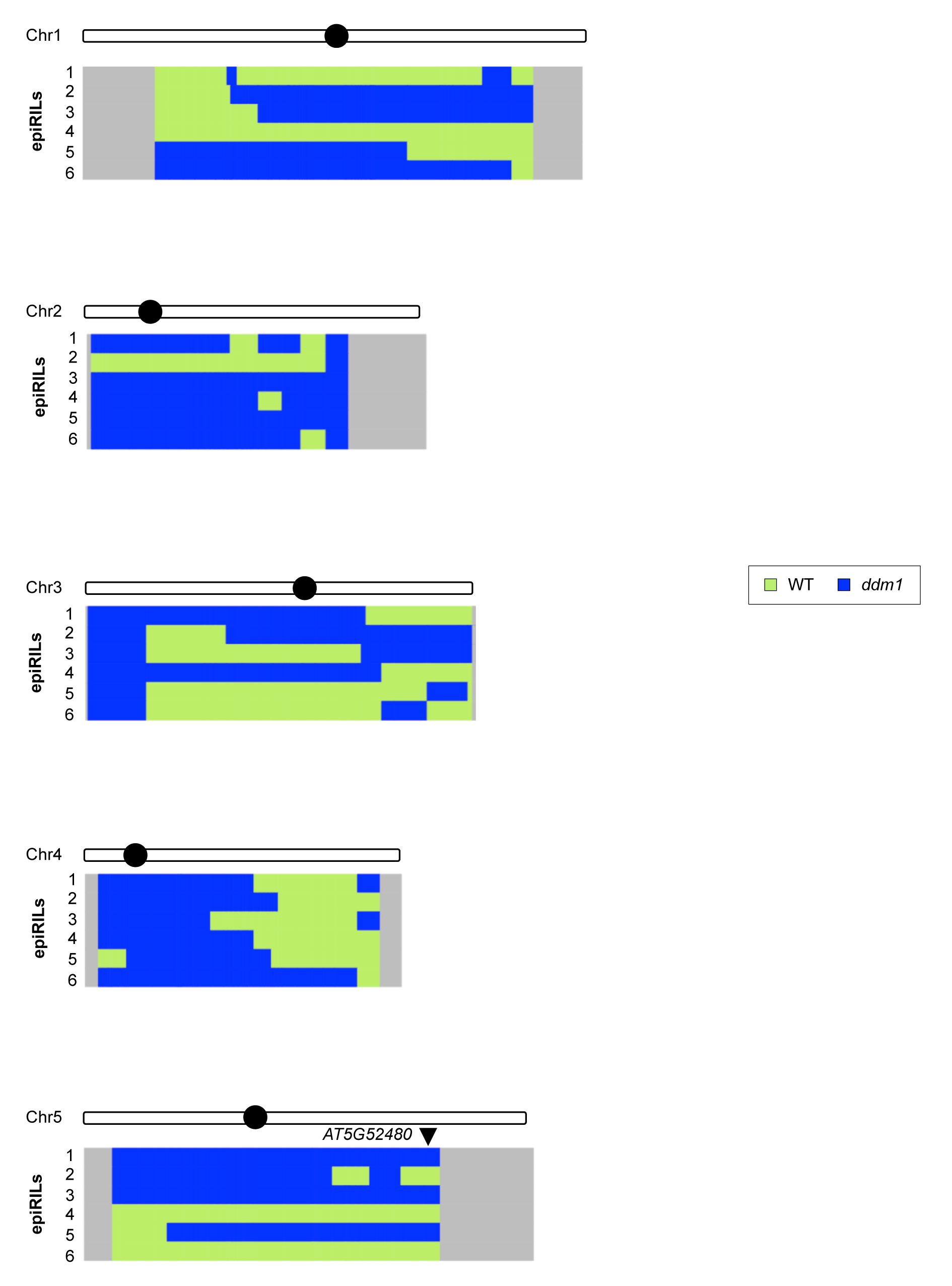

Supplement: S19 Fig — See legend of S18 Fig for details. (TIF) [file pgen.1005154.s021.tif]

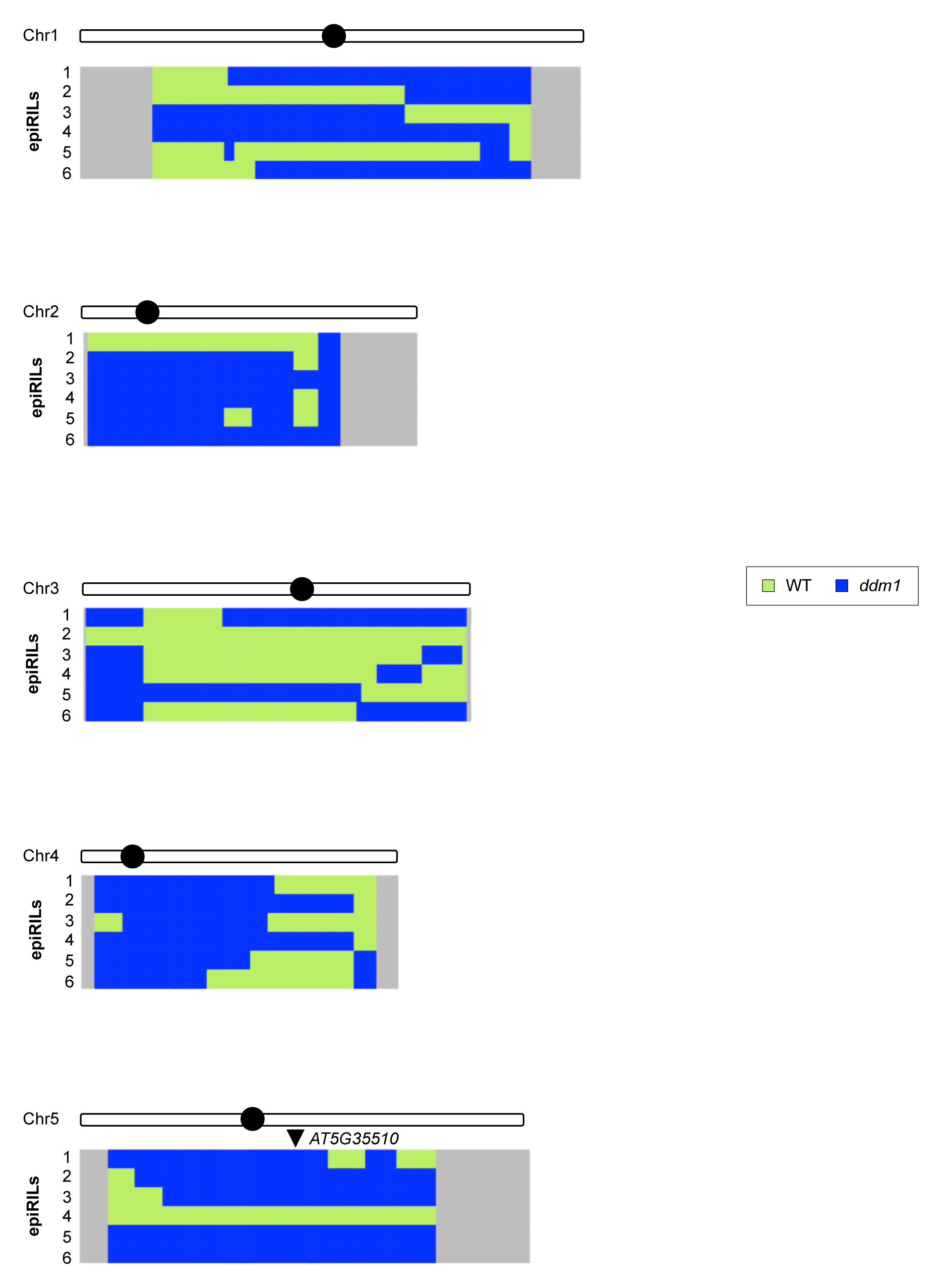

Supplement: S20 Fig — See legend of S18 Fig for details. (TIF) [file pgen.1005154.s022.tif]

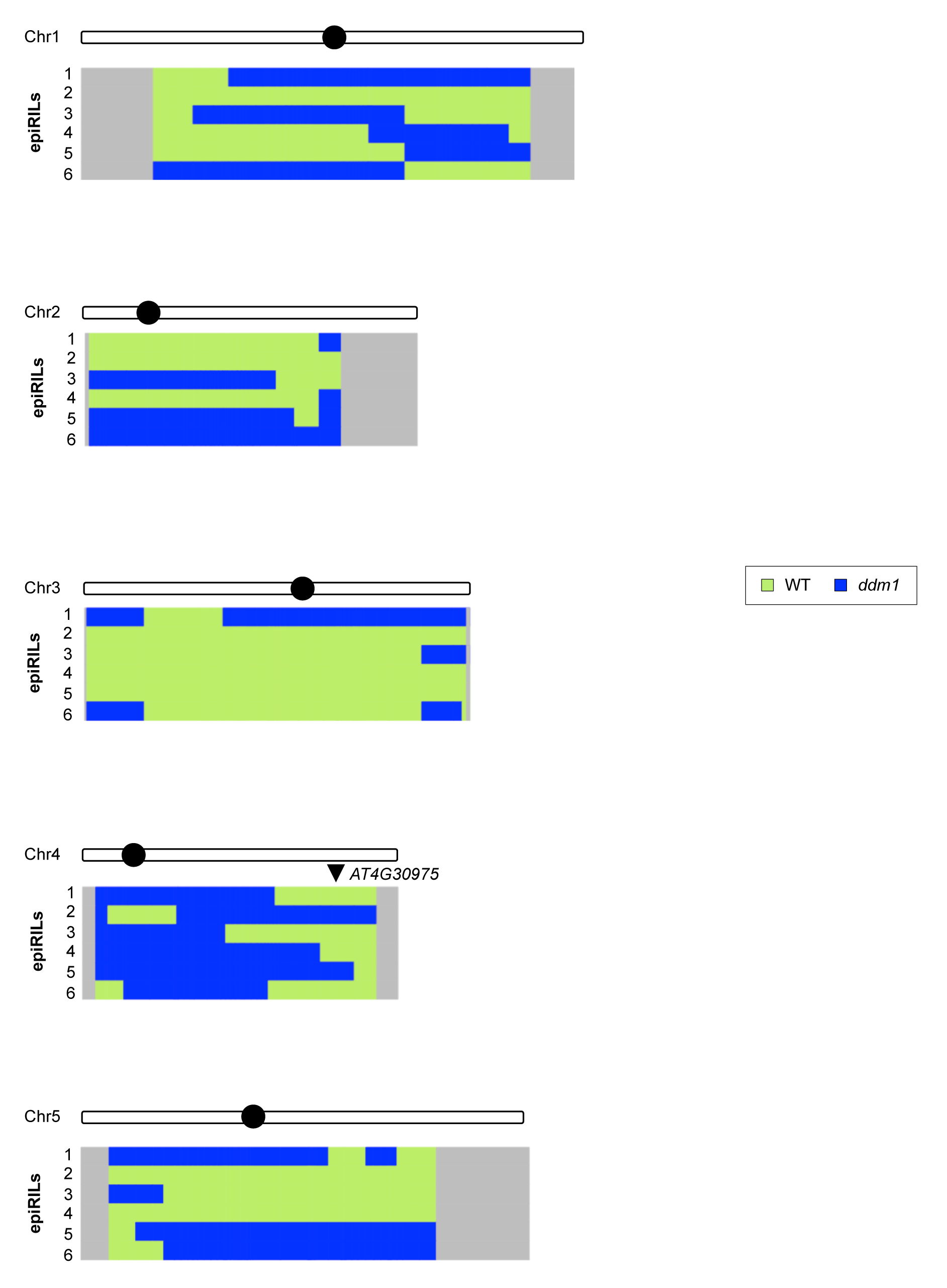

Supplement: S21 Fig — See legend of S18 Fig for details. (TIF) [file pgen.1005154.s023.tif]

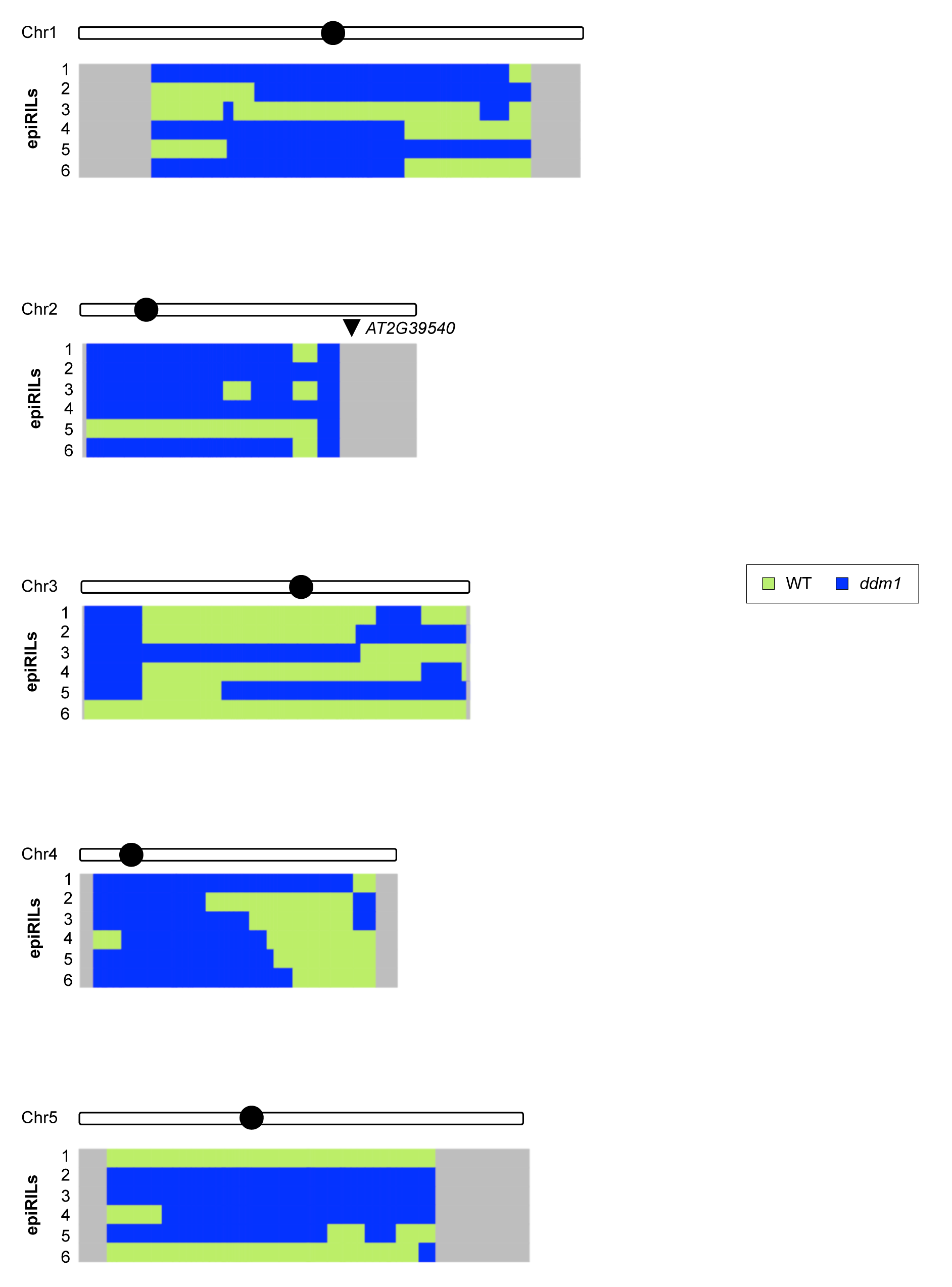

Supplement: S22 Fig — See legend of S18 Fig for details. (TIF) [file pgen.1005154.s024.tif]

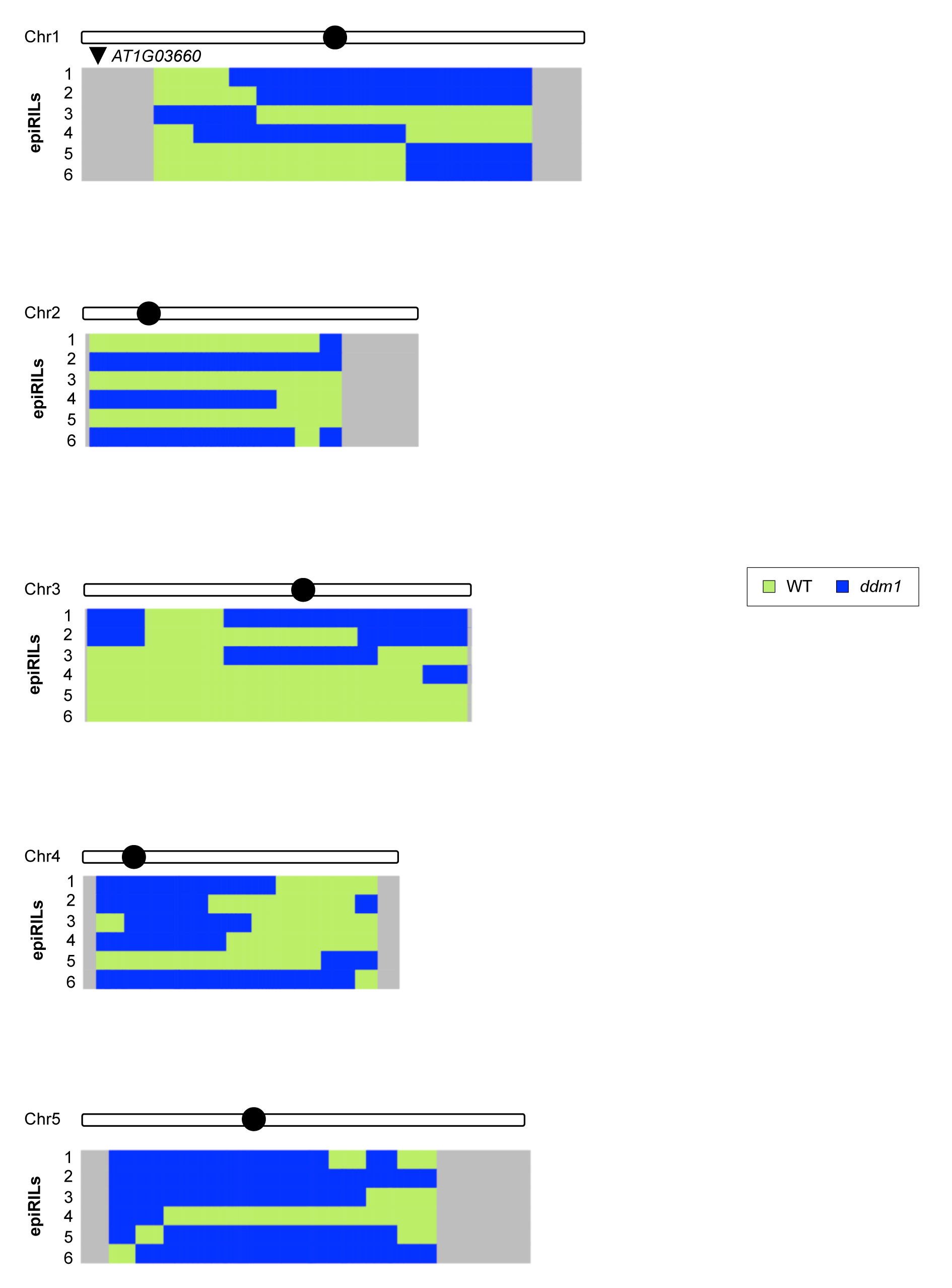

Supplement: S23 Fig — See legend of S18 Fig for details. (TIF) [file pgen.1005154.s025.tif]

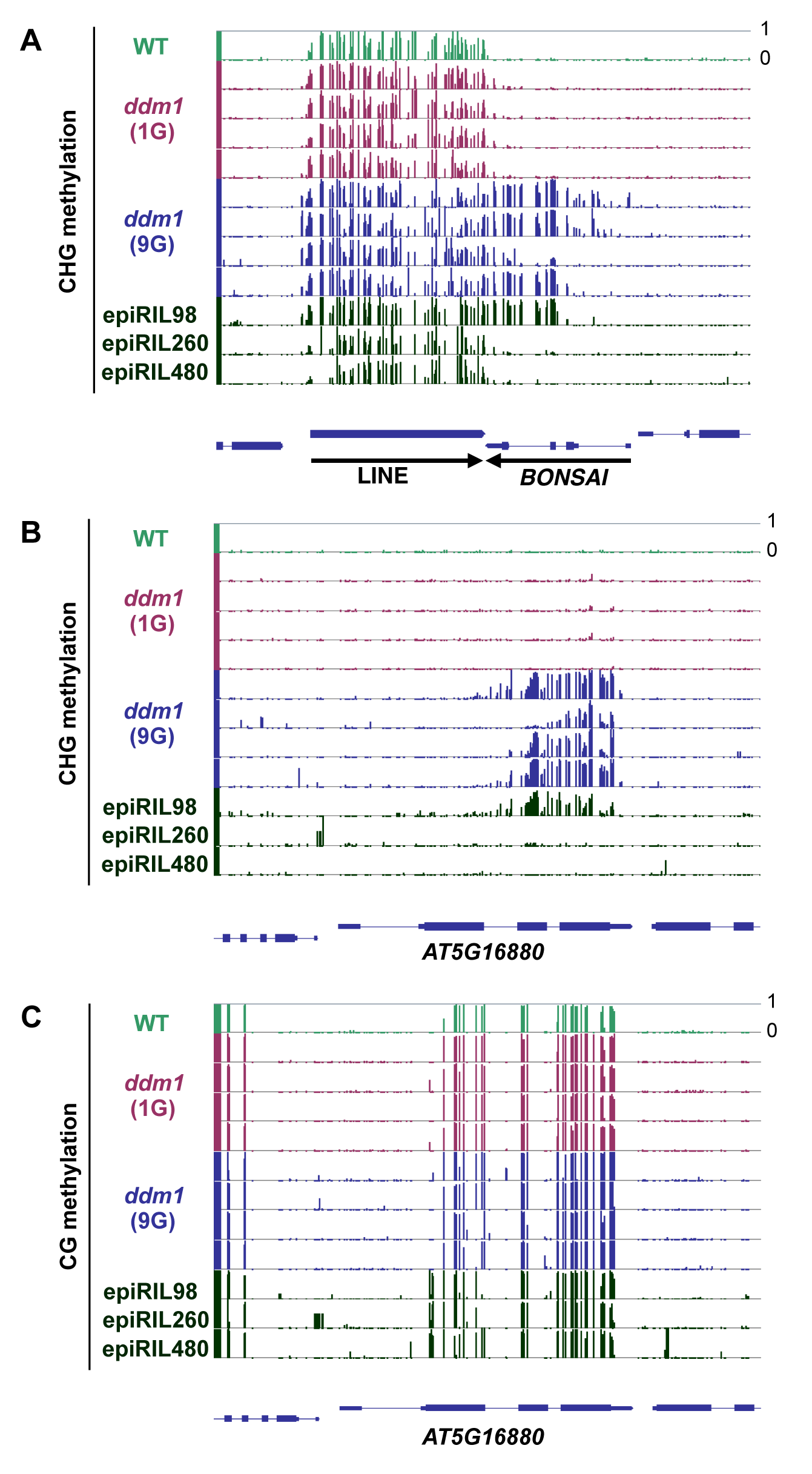

Supplement: S24 Fig — Genome browser views of CHG methylation in the BONSAI (AT1G73177) locus (A), and in AT5G16880 locus (B). The latter locus has a high level of CG methylation (C). For both loci, CHG methylation increased in the 9G ddm1 plants and also in epiRIL98. (TIF) [file pgen.1005154.s026.tif]

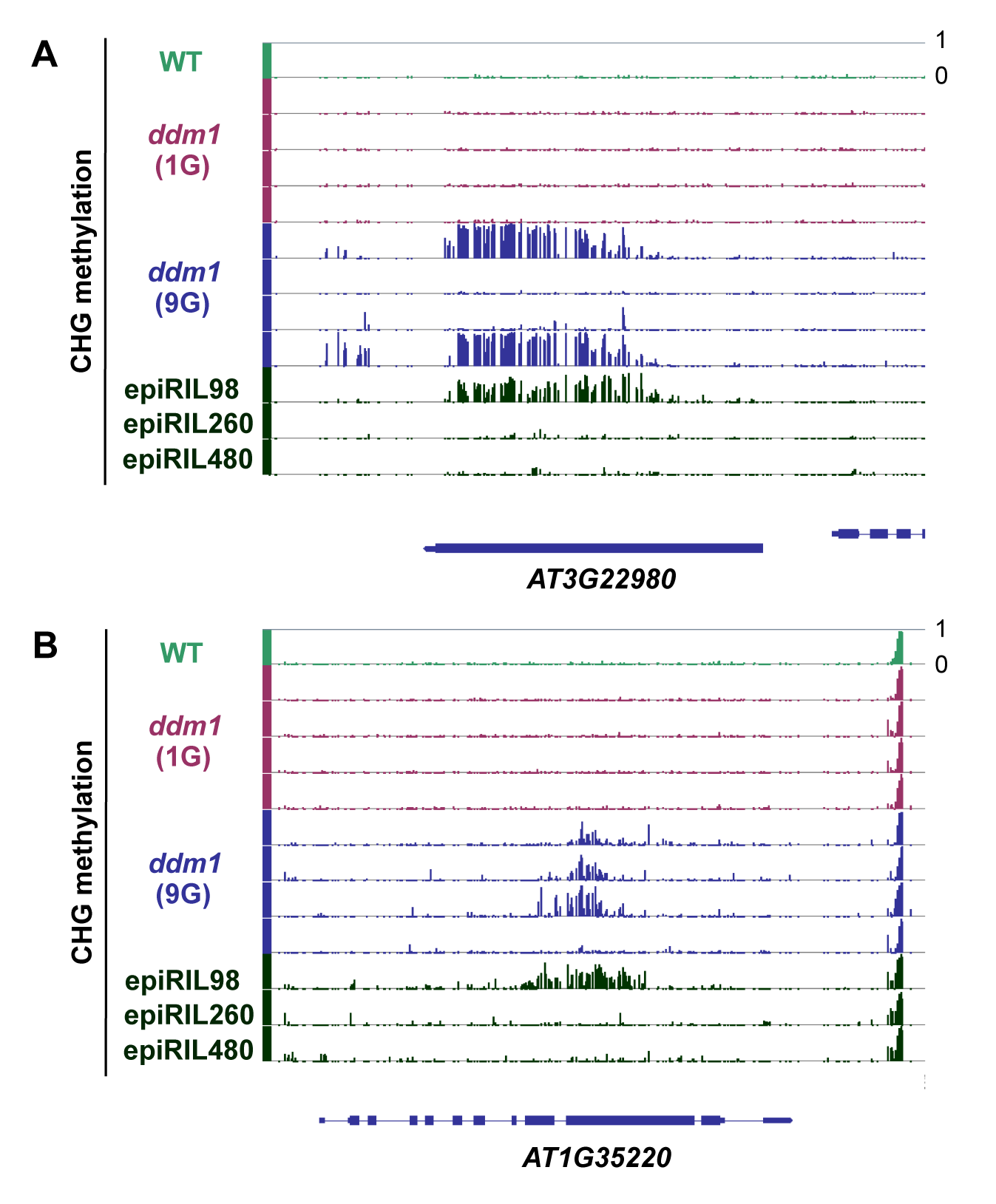

Supplement: S25 Fig — Genome browser views of CHG methylation in AT3G22980 locus (A) and AT1G35220 locus (B). These loci are in the WT-like haplotype in epiRIL98. (TIF) [file pgen.1005154.s027.tif]

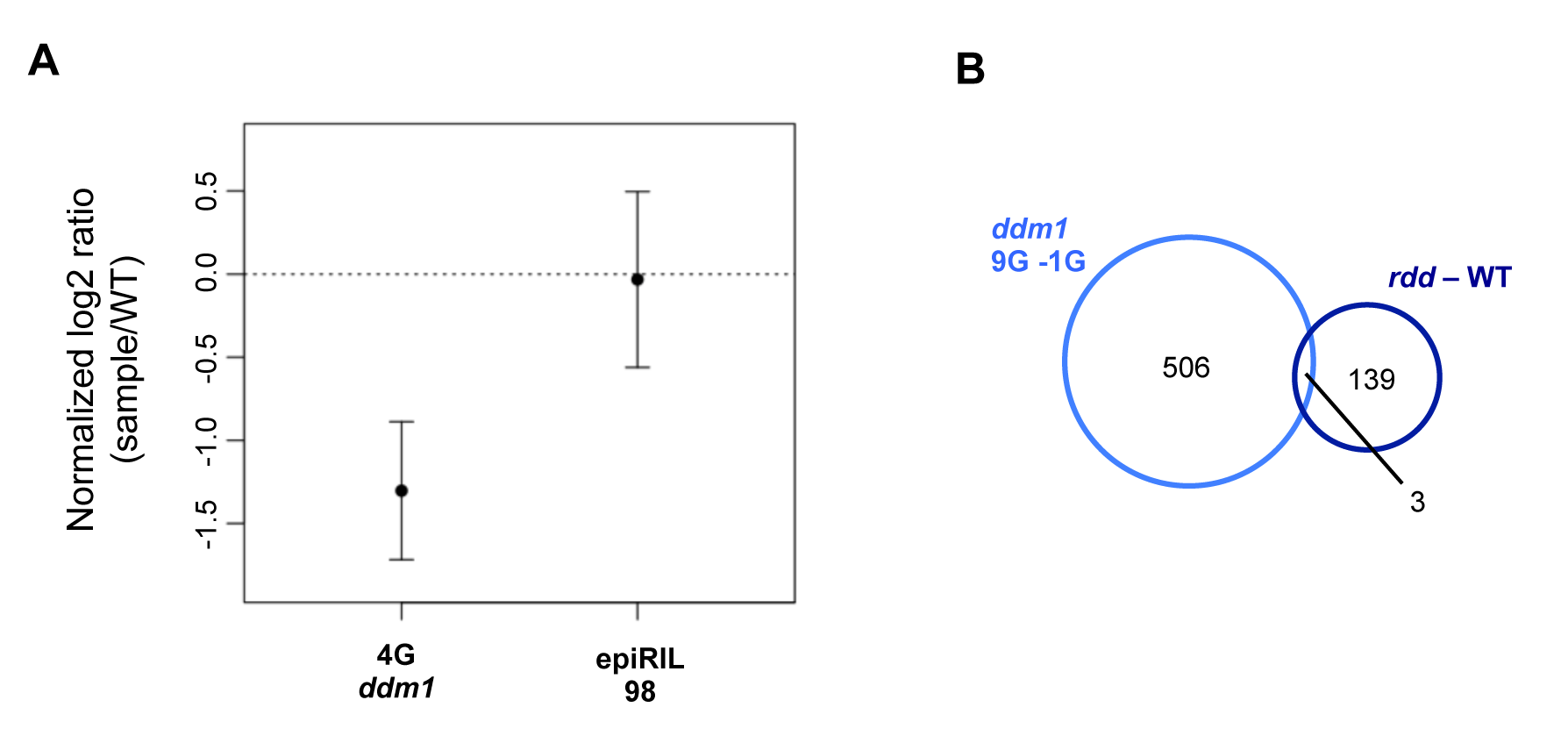

Supplement: S26 Fig — (A) ROS1 gene expression of 4G ddm1, and epiRIL98 compared to WT. Closed circles and error bars indicate the mean and SD of the signals of the probes for ROS1 locus. Although the expression is reduced in the 4G ddm1, it was almost normal for epiRIL98. The data were obtained from GEO (GSE37106 [46]). (B) Overlap between the genes hyper-methylated in ros1-dml2-dml3 triple mutant (data from Penterman et al., 2007 [76]) and the genes hyper-methylated in CHG context during self-pollination of ddm1. (TIF) [file pgen.1005154.s028.tif]

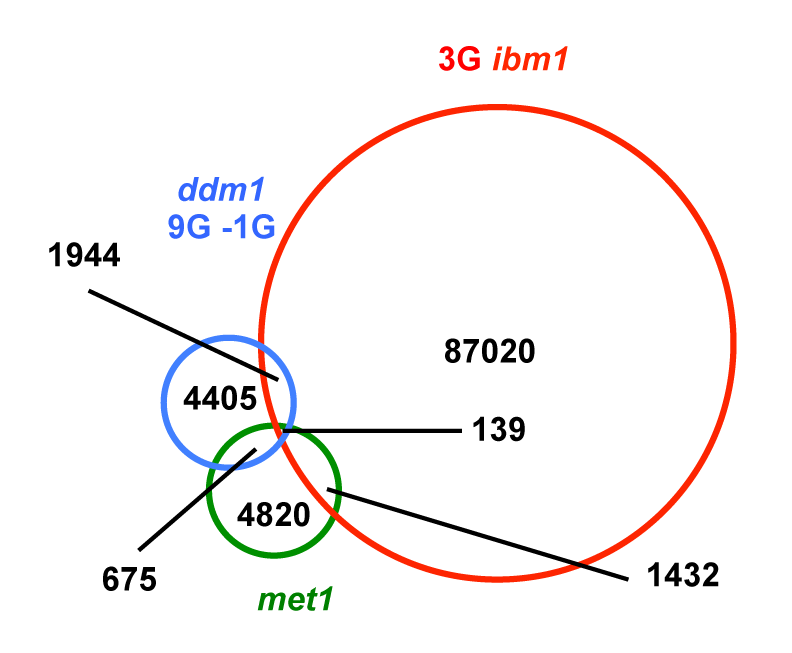

Supplement: S27 Fig — Overlap of regions CHG hyper-methylated in met1, 3G ibm1 and 9G ddm1. DMRs between 9G and 1G ddm1 (blue), between 1G met1 and wild type (green; Data were obtained from GEO (GSE39901 [24]), and between 3G ibm1 and wild type (red) are shown. (TIF) [file pgen.1005154.s029.tif]
